# Multi-class classification of brain tumor using a ResNet101 backbone integrated with multi-scale deformable attention module and advanced data augmentations

**DOI:** 10.1038/s41598-026-45675-y

**Published:** 2026-04-03

**Authors:** B. Sidda Reddy, Ranjeet Ranjan Jha, Abhishek Dasore, Deekshitha Desur, Kiran Shahapurkar, Vineet Tirth, Ali Algahtani, Vijayabhaskara Rao Bhaviripudi, Gezahgn Gebremaryam

**Affiliations:** 1 Department of Mechanical Engineering, Rajeev Gandhi Memorial College of Engineering & Technology, Nandyal, Andhra Pradesh 518501 India; 2 Department of Mathematics, Indian Institute of Technology, Patna, Bihar 801106 India; 3 School of Computer Science and Artificial Intelligence, SR University, Warangal, Telangana 506371 India; 4 Psychiatry Department, Government Medical College, Nizamabad, Telangana 503001 India; 5 Centre of Excellence-Advanced Materials Synthesis (CoE-AMS), Department of Mechanical Engineering, Alliance School of Applied Engineering, Alliance University, Bengaluru, 562106 India; 6 Mechanical Engineering Department, College of Engineering, King Khalid University, 61421 Abha, Aseer Kingdom of Saudi Arabia; 7 Centre for Engineering and Technology Innovations, King Khalid University, 61421 Abha, Aseer Kingdom of Saudi Arabia; 8 Research Center for Advanced Materials Science (RCAMS), King Khalid University, Guraiger, PO Box 9004, 61413 Abha, Aseer Kingdom of Saudi Arabia; 9 Departamento de Física, Facultidad de Ciencias Naturales Matemática y del Medio Ambiente, Universidad Tecnológica Metropolitana, Santiago, Chile; 10 Department of Mechanical Engineering, Wolaita Sodo University, Sodo, Ethiopia

**Keywords:** Multi-class classification, Brain tumor, ResNet101, MS-DAM, Hybrid augmentation, Mixup, Grad-CAM and SHAP analysis, Cancer, Computational biology and bioinformatics, Mathematics and computing, Oncology

## Abstract

Magnetic Resonance Imaging (MRI) scans are crucial role in identifying brain tumors, ensuring accurate clinical diagnosis and effective personalized treatment planning to improve the chances of survival in patients. However, consistent multi-class classification of brain tumours remains a major challenge due to the considerable variability in tumor morphology and the subtle differences among multiple pathological categories. Although there have been tremendous advancements in convolutional neural networks (CNNs) and attention-based deep learning frameworks, challenges remain in achieving robustness performance across multi-class tumor datasets while maintaining interpretability for clinical use. This paper addresses these challenges, by adopting a novel multi-scale deformable attention module (MS-DAM) framework built on ResNet101. The framework is applied on the Kaggle 14-class MRI Brain tumor dataset, to enhance diagnostic accuracy and computational efficiency by capturing the global contextual and local tumor specific features. To improve generalization, hybrid augmentation strategy combined with mixup regularization has been implemented. The explainabiliy of the model is achieved through Grad-CAM and SHAP analyses. The test results of the proposed model are compared with those reported in the existing literature and superior classification accuracy and generalization are observed. The accuracy of the validation and test data set is achieved 96.89% and 99.21% respectively.

## Introduction

 Brain tumor classification from MRI images plays a vital role in clinical diagnosis and treatment planning^1,2^. With the advancement of machine learning and deep learning techniques, particularly convolutional neural networks (CNNs), significant improvements have been achieved in automated tumor detection and classification^3–5^. However, challenges such as tumor heterogeneity, limited annotated data, and high intra-class variability still persist. Early studies employed conventional CNN architectures trained end-to-end for spatial feature extraction. For example, modified AlexNet and custom CNN models achieved promising classification results for glioma and multi-class tumor categorization tasks^6–8^. To address limited medical data, transfer learning approaches using pre-trained architectures such as GoogLeNet, ResNet-50, VGG-16, and EfficientNet were widely adopted^9–12^. Several lightweight and divergence-based CNN variants further improved performance, reporting accuracies above 98%^13–16^. These studies demonstrated the effectiveness of deep CNNs as strong baselines for tumor classification.

Subsequent research focused on improving generalization and representation capability through multi-dataset validation, volumetric learning, patch-based models, and hybrid CNN–Transformer frameworks^17–21^. Multi-branch CNNs, attention-enhanced architectures, Transformer-based models, and comparative analyses of CNNs, Capsule Networks, and Vision Transformers highlighted the growing trend toward multi-scale and attention-based designs^22–26^. Although these models reported high classification performance, many involve increased architectural complexity.

Several works incorporated advanced techniques such as GAN-based pretraining, feature fusion strategies, optimization algorithms, ensemble methods, and hybrid CNN-SVM pipelines to enhance robustness and classification accuracy^27–34^. Traditional handcrafted feature-based approaches, including LBP, clustering, PCA, fuzzy c-means, and level-set methods, were also explored^35–39^. While effective on smaller datasets, these methods relied heavily on manual feature engineering and parameter tuning, limiting their scalability and adaptability to complex tumor variability. Overall, existing approaches demonstrate strong performance; however, challenges related to robustness, generalization across datasets, and efficient feature representation remain open research problems.

### Comparison to deformable attention in prior work

While the term deformable attention has been used in recent MRI tumor classification pipelines, the underlying mechanisms vary substantially. For example, Zarenia et al.^4^. propose a hierarchical multiscale deformable attention module that leverages deformable convolutional networks (DCNs) and forms the attention map by concatenating multi-kernel DCN branches (e.g., 1 × 1, 3 × 3, 5 × 5) followed by pooling and activation, and they employ this module hierarchically within a classification–saliency segmentation pipeline. In contrast, the proposed MS-DAM in this work is a ResNet101-integrated, multi-level feature fusion module where deformability is realized via learned offset prediction and differentiable grid (bilinear) sampling over fused pyramid features (C2–C5), followed by dual attention refinement (spatial attention and channel squeeze-excitation). Accordingly, our MS-DAM differs both architecturally and functionally: it is designed to align and fuse hierarchical backbone features, adaptively resample spatial evidence using offsets, and then refine the representation through complementary spatial and channel attention prior to classification. Building upon this architectural distinction, the overall contribution of the present study extends beyond the attention module itself to the formulation of a unified, end-to-end learning framework.

According to the review of the available literature and to the best of the authors’ knowledge, no prior study has developed a unified, end to end framework for automated multi-class classification of brain tumor using a ResNet101 backbone integrated with a mul-scale deformable attention module and advanced data augmentations. Existing approaches typically focus on isolated components such as conventional CNN architectures, transfer learning strategies, or standalone attention mechanisms. To address these limitations, the proposed framework combines adaptive feature learning, hybrid data augmentation using MixUp, and optimization strategies to improve robustness against class imbalance and MRI heterogeneity. Furthermore, model interpretability is supported through Grad-CAM and SHAP to provide clinically meaningful insights into the decision-making process.

## Methodology

**Fig. 1:**
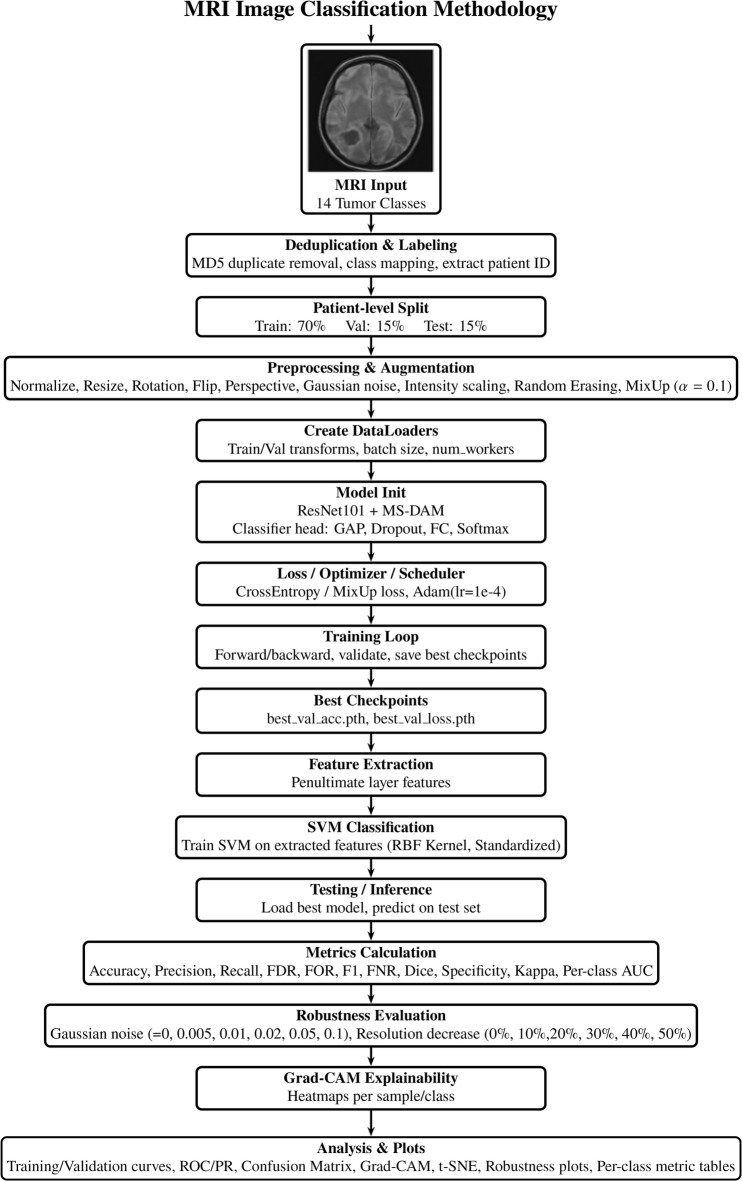
Brain tumor classification methodology.

The proposed system integrates a ResNet101 backbone with a Multi-Scale Deformable Attention Module (MS-DAM) to effectively capture both global semantic information and local spatial variations within heterogeneous tumor regions. The ResNet101 architecture is chosen as the backbone because of its deep residual learning framework, which enables stable gradient propagation and effective extraction of high-level features from medical images. Compared with shallower networks such as VGG16 or computationally heavier architectures such as DenseNet and EfficientNet variants, ResNet101 provides a balanced trade-off between representational depth, training stability, and computational efficiency. Furthermore, the use of a well-established backbone allows the study to focus on evaluating the effectiveness of the proposed MS-DAM module while maintaining fair comparison with prior medical imaging studies. This hybrid architecture enables adaptive multi-scale feature fusion and spatial attention modeling, enhancing the model’s ability to distinguish between different types of tumors. The complete methodology adopted for brain tumor classification is shown in Fig. 1.

### Dataset preparation

#### Dataset scanning and deduplication

The dataset used in the present research contains 15 classes of MRI brain turmor images including normal (non-tumorous) images. Most patients contribute a single image, and patient-level splitting ensures independence across datasets. The dataset directory from kagglehub.dataset_download (“waseemnagahhenes/brain-tumor-for-14-classes”)^40^ is recursively scanned to detect all image files. To ensure integrity all images are hashed for removing duplicates using the MD5 hash function:

1 $$\:{\mathrm{h}}_{\mathrm{i}}=\:\mathrm{M}\mathrm{D}5({\mathrm{i}\mathrm{m}\mathrm{a}\mathrm{g}\mathrm{e}}_{\mathrm{i}},\:\mathrm{t}\mathrm{o}\mathrm{b}\mathrm{y}\mathrm{t}\mathrm{e}\mathrm{s}(\left)\right)$$

where, $\:{image}_{i}$ denotes the i^th^ MRI image tensor considered after RGB conversion, and $\:{h}_{i}$ is the 128-bit hash signature used to detect and remove identical images.

Only unique hashes are retained, confirming that visually identical images are not reused in either training and validation sets.

Each image is the labelled using its parent directory name (representing the class), and a patient ID is extracted from the filename pattern < pid> _…….

2 $$\:\mathrm{p}\mathrm{i}\mathrm{d}\:=\:\mathrm{s}\mathrm{p}\mathrm{l}\mathrm{i}\mathrm{t}\left(\mathrm{s}\mathrm{t}\mathrm{e}\mathrm{m} (\mathrm{I}\mathrm{i}),\:\mathrm{`} \_ \mathrm{''} \right)\left[0\right]$$

To further ensure robustness against subtle duplication artifacts, the MD5 hash was computed on the full RGB pixel tensor after conversion and normalization. Images producing identical 128-bit hash signatures were considered exact duplicates and removed prior to dataset partitioning. This guarantees that no identical MRI scan appears across different subsets.

To lessen the impact of class imbalance across tumor groups, numerous strategies were implemented. First, data augmentation methods were employed to the training set to raise sample diversity and enhance minority-class representation. Second, MixUp regularization was applied during training to produce interpolated samples between classes, which aids stabilize decision boundaries and expands generalization across imbalanced categories. Finally, model performance was assessed using class-balanced metrics such as precision, recall, and F1-score to confirm fair evaluation across all tumor classes.

#### Patient wise data split

To prevent data leakage between scans of the same object, all images form the same patient were grouped together and assigned to one subset only. Let $\:P$ is the set of unique IDs, then:

$$\:{P}_{train\:}\bigcap\:\:{P}_{val}=0.\:{P}_{train\:}\bigcap\:\:{P}_{test}=0.\:\:{P}_{val\:}\bigcap\:\:{P}_{test}=0$$

The unique samples are usually split into 70% for training, 15% for validation and 15% for testing.

To quantitatively verify strict patient-level isolation, we computed the total number of samples and unique patient IDs per split after partitioning. The resulting distribution was:

Training: 3318 images (2275 unique patients).

Validation: 579 images (487 unique patients).

Testing: 508 images (488 unique patients).

Set intersection operations were explicitly performed between the three patient ID groups, and zero overlap was confirmed in all cases. This programmatic verification ensures that no subject-level information is shared across training, validation, and testing subsets.

The dataset was partitioned strictly at the patient level (70%/15%/15%) prior to model training, and the test set remained completely unseen during model optimization.

### Data augmentation and normalization

Due to scanner noise, head orientation, and illumination inconsistencies, MRI scans often exhibit variations. To improve model generalization, robustness and prevents overfitting, data augmentation introduces artificial variability to mimic the real-world conditions.

The transformation function T(⋅) applied to each image_i_ (I) is represented as:

3 $$\begin{aligned}\mathrm{I}^{\prime\:}=\mathrm{T}\left(\mathrm{I}\right) &=\mathrm{R}\mathrm{a}\mathrm{n}\mathrm{d}\mathrm{o}\mathrm{m}\mathrm{R}\mathrm{e}\mathrm{s}\mathrm{i}\mathrm{z}\mathrm{e}\mathrm{d}\mathrm{C}\mathrm{r}\mathrm{o}\mathrm{p}\circ\:\mathrm{R}\mathrm{a}\mathrm{n}\mathrm{d}\mathrm{o}\mathrm{m}\mathrm{F}\mathrm{l}\mathrm{i}\mathrm{p}\mathrm{H},\mathrm{V} \\ & \quad \circ\:\mathrm{R}\mathrm{a}\mathrm{n}\mathrm{d}\mathrm{o}\mathrm{m}\mathrm{R}\mathrm{o}\mathrm{t}\mathrm{a}\mathrm{t}\mathrm{i}\mathrm{o}\mathrm{n}\circ\:\mathrm{P}\mathrm{e}\mathrm{r}\mathrm{s}\mathrm{p}\mathrm{e}\mathrm{c}\mathrm{t}\mathrm{i}\mathrm{v}\mathrm{e}\circ\:\mathrm{A}\mathrm{d}\mathrm{d}\mathrm{G}\mathrm{a}\mathrm{u}\mathrm{s}\mathrm{s}\mathrm{i}\mathrm{a}\mathrm{n}\mathrm{N}\mathrm{o}\mathrm{i}\mathrm{s}\mathrm{e} \\ & \quad \circ\:\:\mathrm{R}\mathrm{a}\mathrm{n}\mathrm{d}\mathrm{o}\mathrm{m}\mathrm{I}\mathrm{n}\mathrm{t}\mathrm{e}\mathrm{n}\mathrm{s}\mathrm{i}\mathrm{t}\mathrm{y}\mathrm{S}\mathrm{c}\mathrm{a}\mathrm{l}\mathrm{i}\mathrm{n}\mathrm{g}\:\circ\:\:\mathrm{C}\mathrm{o}\mathrm{l}\mathrm{o}\mathrm{r}\mathrm{J}\mathrm{i}\mathrm{t}\mathrm{t}\mathrm{e}\mathrm{r}\circ\:\:\mathrm{R}\mathrm{a}\mathrm{n}\mathrm{d}\mathrm{o}\mathrm{m}\mathrm{E}\mathrm{r}\mathrm{a}\mathrm{s}\mathrm{i}\mathrm{n}\mathrm{g}\left(\mathrm{I}\right)\end{aligned}$$

where:

- ∘ denotes functional composition (operations applied sequentially).
- RandomResizedCrop randomly crops the image and rescales to 224 × 224.
- RandomFlip_H, V_ denotes horizontal and vertical flips with probability *p* = 0.5.
- AddGaussianNoise adds Gaussian perturbations with mean 0 and variance σ^2^.
- RandomErasing removes small regions of the image to simulate occlusions.

For validation and testing, deterministic transformations are applied:

$$\:\mathrm{I}{\prime\:}=\mathrm{R}\mathrm{e}\mathrm{s}\mathrm{i}\mathrm{z}\mathrm{e}(\mathrm{I},224\times\:224)\to\:\mathrm{N}\mathrm{o}\mathrm{r}\mathrm{m}\mathrm{a}\mathrm{l}\mathrm{i}\mathrm{z}\mathrm{e}\left(\mathrm{I}\right)\:$$

Normalization uses the ImageNet mean (µ) and standard deviation (σ):

4 $$\:{I}^{"}=\:\frac{{I}^{{\prime\:}}-\mu\:}{\sigma\:}$$

### MixUp data augmentation

MixUp is a linear interpolation between random pairs of images and their labels

5 $$\:\stackrel{\sim}{x}=\lambda\:{x}_{i}+\left(1-\lambda\:\right){x}_{j}$$

6 $$\:\stackrel{\sim}{y}=\lambda\:{y}_{i}+(1-\lambda\:){y}_{j}$$

7 $$\:\lambda\:\sim Beta\left(\alpha\:,\alpha\:\right),\:\alpha\:=0.1$$

The training loss becomes

8 $$\:{L}_{mixup}=\lambda\:CE\left(f(\stackrel{\sim}{x}\right),{y}_{i})\:+\left(1-\lambda\:\right)CE\left(f(\stackrel{\sim}{x}\right),{y}_{j})\:$$

### Model architecture

#### ResNet101 backbone

A pretrained ResNet101 is used as the base network to serve as backbone to extract hierarchical multi-scale feature maps, from its convolutional layers. The ResNet101 built on residual learning principles.

Each residual block learns a mapping

9 $$\:H\left(x\right)\:=\:F(x,\:{w}_{i})\:+\:x$$

Where $\:F(x,\:{w}_{i})\:$ is the learned residual function parameterized by weights $\:{w}_{i}$, (i.e., residual mapping with convolutional weights). This allows the network to learn residual features efficiently and $\:x$ is the identity shortcut connection.

The backbone extracts hierarchical features:

10 $$\:\{\mathrm{C}2,\:\mathrm{C}3,\:\mathrm{C}4,\:\mathrm{C}5\}\:=\:\mathrm{R}\mathrm{e}\mathrm{s}\mathrm{N}\mathrm{e}\mathrm{t}101\left(\mathrm{X}\right)\:=\:\{\mathrm{l}\mathrm{a}\mathrm{y}\mathrm{e}\mathrm{r}1,\:\mathrm{l}\mathrm{a}\mathrm{y}\mathrm{e}\mathrm{r}2,\:\mathrm{l}\mathrm{a}\mathrm{y}\mathrm{e}\mathrm{r}3,\:\mathrm{l}\mathrm{a}\mathrm{y}\mathrm{e}\mathrm{r}4\}$$

Where $\:{C}_{i}$ denotes feature maps of increasing receptive field size.

$$\:{C}_{2}:\:256,\:{C}_{3}:\:512,\:{C}_{4}:\:1024,\:{C}_{5}:\:2048$$ channels.

#### Multi-scale deformable attention module (MS-DAM)

The proposed hybrid framework integrates ResNet101 as a backbone with a MS-DAM and introduces learnable deformable attention. ResNet101, with 101 layers, utilizes residual connections to mitigate vanishing gradients and allows training of deep neural networks.

This refines feature fusion across multiple scales by applying learnable deformable offsets and dual (spatial + channel) attention. It overcomes the fixed receptive field limitation of standard convolution by dynamically sampling informative spatial locations.

Given each input feature maps $\:{f}_{i}=\:{\mathbb{R}}^{B\times\:{C}_{i}\times\:H\times\:{W}_{i}}$, the operations are:

- *Projection to uniform dimension*.
  $$\:{p}_{i}=\:\mathrm{R}\mathrm{e}\mathrm{L}\mathrm{U}\left(\mathrm{B}\mathrm{N}\right(\mathrm{C}\mathrm{o}\mathrm{n}\mathrm{v}1\times\:1\left({\mathrm{f}}_{\mathrm{i}}\right)\left)\right)$$
  where, $\:{Conv}_{1\times\:1}\:=1\times\:1$ convolution for channel compression,
  BN: batch normalization.
  ReLU: Rectified linear unit activation.
- *Spatial Upsampling*.
  All projected maps $\:{p}_{i}$ are bilinearly interpolated to the same spatial resolution i.e., reference size $\:\left({H}_{r},{\:W}_{r}\right)$ as the largest map.
  11 $$\:{p}_{i}^{{\prime\:}}=Interpolate({p}_{i},\:\left({H}_{r},{\:W}_{r}\right))$$
- *Concatenation*.
  12 $$\:{F}_{c}=[{p}_{1}^{{\prime\:}},{p}_{2}^{{\prime\:}},{p}_{3}^{{\prime\:}}\dots\:\dots\:.{p}_{n}^{{\prime\:}}]$$
- *Offset prediction*.
  The deformable mechanism learns spatial offsets $\:\varDelta\:\:=({\varDelta\:}_{x},{\varDelta\:}_{y})$ for each sampling point. Offsets re predicted by light weight convolutional sub-network.
  13 $$\:\varDelta\:\:=s.\mathrm{tanh}\left({Conv}_{3\times\:3}\left({F}_{c}\right)\right)$$
  Where $\:s\:=$ scaling factor typically 0.12 controlling maximum displacement,
  tanh bounds the offsets to $\:(-1,\:1)$.
- *Deformable sampling grid construction*.
  A base or regular grid in normalized coordinates is defined as.
  14 $$\:{x}_{base}\left(j\right)=\:-1+\:\frac{2j}{W-1};\:{y}_{base}\left(i\right)=\:-1+\:\frac{2i}{H-1}$$
  Forming $$\:{G}_{base}(i,\:j)\:=\:({x}_{base},\:{y}_{base})$$.
  The final Deformable sampling grid becomes:
  15 $$\:{G}_{sample}(i,\:j)\:=\:clamp\left({G}_{base}\right(i,\:j)\:+\:\varDelta\:(i,\:j),\:-1,\:1)\:$$
  Each coordinate in $\:{G}_{sample}$ defines a position from which the feature map is sampled using bilinear interpolation.
- *Bilinear sampling equation*.
  For each sampling point, i.e. for a target coordinate $\:({x}_{p},\:{y}_{p})$.
  16 $$\:v\left({x}_{p},\:{y}_{p}\right)=\left(1-u\right)\left(1-v\right)F\left({x}_{0},\:{y}_{0}\right)+u\left(1-v\right)F\left({x}_{1},\:{y}_{0}\right)+\left(1-u\right)vF\left({x}_{0},\:{y}_{1}\right)+uvF({x}_{1},\:{y}_{1})$$
  Where $\:\left({x}_{0},\:{y}_{0}\right)$, $\:\left({x}_{1},\:{y}_{0}\right)$, $\:\left({x}_{0},\:{y}_{1}\right)$, $\:({x}_{1},\:{y}_{1})$are integer neighbours around $\:\left({x}_{p},\:{y}_{p}\right)$,
  17 $$\:u={x}_{p}-\left|{x}_{p}\right|,\:v={y}_{p}-\left|{y}_{p}\right|$$
  the sampled value $\:v\left({x}_{p},\:{y}_{p}\right)$ is differentiable with respect to both the input $\:F\:$and the offsets $\:\varDelta\:$.
- *Gradient propagation*.
  During back propagation, partial derivatives are propagated as:
  18 $$\:\frac{dV({x}_{p},\:{y}_{p})}{d{x}_{p}}=-\left(1-v\right)F\left({x}_{o},\:{y}_{o}\right)+\left(1-v\right)F\left({x}_{1},\:{y}_{0}\right)-vF\left({x}_{0},\:{y}_{1}\right)+vF\left({x}_{1},{y}_{1}\right)$$
  Thus, allowing the offset predictor to learn optimal spatial displacements.
- *Attention Mechanisms*.
  After deformable sampling, the fused features are reshaped and averaged $\:{F}_{m}$ and passes through attention blocks.
  19 $$\:{F}_{m}=\frac{1}{n}\sum\:_{i=1}^{n}{F}_{i}^{sampled}$$
- *Spatial attention*:
  20 $$\:{A}_{s}=\:\sigma\:\left({Con}_{1\times\:1}\left(ReLU\left({Con}_{3\times\:3}\left({F}_{m}\right)\right)\right)\right)$$
  where $\:\sigma\:$ is the sigmoid activation mapping the spatial mask to [0, 1].
  Channel (squeeze-excitation) attention:$\:\:{A}_{s}=\:\sigma\:\left({W}_{2}\left(ReLU\left({W}_{1}(GAP\left({F}_{m}\right)\right)\right)\right)$
  GAP: Global Average pooling over spatial dimensions,
  W_1_, W_2_: $$\:1\times\:1$$ convolution layers.
- *Attention weighted feature refinement and fusion*.
  21 $$\:{F}_{fused}=\:ReLU\left({BN(Con}_{3\times\:3}\left({A}_{s\:\:}\odot\:\left({A}_{c\:\:}\odot\:{F}_{m\:\:}\right)\right)\right))$$
  Where $\:\odot\:$ element wise multiplication.
  Classification layer or head:
  The final fused feature is globally averaged, regularized with dropout and classified:
  22 $$\:Y\:=\:Softmax\left(FC\right(GAP\left({F}_{fused}\right)\left)\right)$$
- *Distinction from DCN-based multiscale deformable attention (Zarenia et al.*^4^)
  It is important to note that our MS-DAM is not a DCN multi-kernel concatenation attention block. Unlike Zarenia et al.^4^, where deformability is primarily embedded inside DCN branches (with different kernel sizes) and the attention map is produced from concatenated DCN outputs, our deformability is implemented through an explicit offset-grid sampling formulation. We first fuse multi-level backbone features (C2–C5) via projection, resolution alignment, and concatenation; then we predict bounded offsets and construct a deformable sampling grid to adaptively sample informative spatial locations using differentiable bilinear interpolation. The sampled fused representation is subsequently refined using dual attention-a spatial attention mask to emphasize tumor-relevant locations and a channel squeeze-excitation pathway to recalibrate inter-channel dependencies. This design specifically targets multi-scale MRI heterogeneity by combining (i) hierarchical feature fusion, (ii) deformable resampling at the fusion level, and (iii) complementary spatial+channel attention, which collectively establish the methodological novelty of the proposed MS-DAM.

### Training strategy

Training minimizes the cross-entropy loss between predicted class probabilities and ground-truth labels

23 $$\:LCE=-\sum\:_{i=1}^{C}{y}_{i}log\left({\widehat{y}}_{i}\right)$$

Where $\:{\widehat{y}}_{i}$ is the predicted probability for class $\:i$.

If MixUP is used the modified loss $\:{L}_{mixup}$ is applied.

Optimizer: Adam.

24 $$\:{W}_{t+1}={W}_{t}-\eta\:\frac{{\widehat{m}}_{t}}{\sqrt{{\widehat{v}}_{t}}+\varepsilon}\:\:$$

Where $\:{\widehat{m}}_{t}$ and $\:{\widehat{v}}_{t}$ are bias-corrected first and second moment estimates, $\:\eta\:=1\times\:{10}^{-4}$ and $\varepsilon=\:{10}^{-8}$.

### Evaluation metrics

- *Accuracy*: accuracy means the proportion of correct predictions out of all prediction:
  $$\:\mathrm{A}\mathrm{c}\mathrm{c}\mathrm{u}\mathrm{r}\mathrm{a}\mathrm{c}\mathrm{y}=\:\frac{\mathrm{N}\mathrm{o}.\:\mathrm{o}\mathrm{f}\:\mathrm{c}\mathrm{o}\mathrm{r}\mathrm{r}\mathrm{e}\mathrm{c}\mathrm{t}\:\mathrm{p}\mathrm{r}\mathrm{e}\mathrm{d}\mathrm{i}\mathrm{c}\mathrm{t}\mathrm{i}\mathrm{o}\mathrm{n}\mathrm{s}}{\mathrm{T}\mathrm{o}\mathrm{t}\mathrm{a}\mathrm{l}\:\mathrm{p}\mathrm{r}\mathrm{e}\mathrm{d}\mathrm{i}\mathrm{c}\mathrm{t}\mathrm{i}\mathrm{o}\mathrm{n}\mathrm{s}}$$
- *Macro Average*: these are ways to aggregate metrics (like Precision, recall F1-score etc.) across multiple classes.
  $$\:\mathrm{M}\mathrm{a}\mathrm{c}\mathrm{r}\mathrm{o}\:\mathrm{A}\mathrm{v}\mathrm{e}\mathrm{r}\mathrm{a}\mathrm{g}\mathrm{e}=\:\frac{1}{\mathrm{C}}\sum\:_{\mathrm{i}=1}^{\mathrm{C}}{\mathrm{M}\mathrm{e}\mathrm{t}\mathrm{r}\mathrm{i}\mathrm{c}}_{\mathrm{i}}$$
  Treats all classes equally, regardless of how many samples each class has.
- *Micro average*: Compute the global metric across all samples, ignoring class boundaries. Heavily influenced by majority classes.
  $$\:\mathrm{M}\mathrm{i}\mathrm{c}\mathrm{r}\mathrm{o}\:\mathrm{A}\mathrm{v}\mathrm{e}\mathrm{r}\mathrm{a}\mathrm{g}\mathrm{e}=\:\frac{\mathrm{S}\mathrm{u}\mathrm{m}\:\mathrm{o}\mathrm{f}\:\mathrm{T}\mathrm{r}\mathrm{u}\mathrm{e}\:\mathrm{P}\mathrm{o}\mathrm{s}\mathrm{i}\mathrm{t}\mathrm{i}\mathrm{v}\mathrm{e}\mathrm{s}}{\mathrm{T}\mathrm{o}\mathrm{t}\mathrm{a}\mathrm{l}\:\mathrm{S}\mathrm{a}\mathrm{m}\mathrm{p}\mathrm{l}\mathrm{e}\mathrm{s}}$$
- *Precision (Positive predictive Value)*.
  $$\:\mathrm{P}\mathrm{r}\mathrm{e}\mathrm{c}\mathrm{i}\mathrm{s}\mathrm{i}\mathrm{o}\mathrm{n}=\:\frac{\:\mathrm{T}\mathrm{r}\mathrm{u}\mathrm{e}\:\mathrm{P}\mathrm{o}\mathrm{s}\mathrm{i}\mathrm{t}\mathrm{i}\mathrm{v}\mathrm{e}\mathrm{s}}{\mathrm{T}\mathrm{r}\mathrm{u}\mathrm{e}\:\mathrm{P}\mathrm{o}\mathrm{s}\mathrm{i}\mathrm{t}\mathrm{i}\mathrm{v}\mathrm{e}\mathrm{s}+\mathrm{F}\mathrm{a}\mathrm{l}\mathrm{s}\mathrm{e}\:\mathrm{P}\mathrm{o}\mathrm{s}\mathrm{i}\mathrm{t}\mathrm{i}\mathrm{v}\mathrm{e}\mathrm{s}}$$
- *Recall (Sensitivity/True Positive Rate)*:
  $$\:R\mathrm{e}\mathrm{c}\mathrm{a}\mathrm{l}\mathrm{l}\:=\:\frac{\:\mathrm{T}\mathrm{r}\mathrm{u}\mathrm{e}\:\mathrm{P}\mathrm{o}\mathrm{s}\mathrm{i}\mathrm{t}\mathrm{i}\mathrm{v}\mathrm{e}\mathrm{s}}{\mathrm{T}\mathrm{r}\mathrm{u}\mathrm{e}\:\mathrm{P}\mathrm{o}\mathrm{s}\mathrm{i}\mathrm{t}\mathrm{i}\mathrm{v}\mathrm{e}\mathrm{s}+\mathrm{F}\mathrm{a}\mathrm{l}\mathrm{s}\mathrm{e}\:\mathrm{N}\mathrm{e}\mathrm{g}\mathrm{a}\mathrm{t}\mathrm{i}\mathrm{v}\mathrm{e}\mathrm{s}}$$
  It shows, how many actual positives the model correctly identified.
- *F1-score*: It shows, balance between precision and recall.
  $$\:\mathrm{F}1\:=\:2\frac{\:\mathrm{P}\mathrm{r}\mathrm{e}\mathrm{c}\mathrm{i}\mathrm{s}\mathrm{i}\mathrm{o}\mathrm{n}\:\times\:\mathrm{R}\mathrm{e}\mathrm{c}\mathrm{a}\mathrm{l}\mathrm{l}}{\mathrm{P}\mathrm{r}\mathrm{e}\mathrm{c}\mathrm{i}\mathrm{s}\mathrm{i}\mathrm{o}\mathrm{n}+\mathrm{R}\mathrm{e}\mathrm{c}\mathrm{a}\mathrm{l}\mathrm{l}}$$
- Dice Coefficient (F1 variant for segmentation).
  $$\:\mathrm{D}\mathrm{i}\mathrm{c}\mathrm{e}\:=\:\frac{2\:\mathrm{T}\mathrm{P}}{2\mathrm{T}\mathrm{P}+\mathrm{F}\mathrm{P}+\mathrm{F}\mathrm{N}}$$
- Specificity (True Negative Rate):
  $$\:\mathrm{S}\mathrm{p}\mathrm{e}\mathrm{c}\mathrm{i}\mathrm{f}\mathrm{i}\mathrm{c}\mathrm{i}\mathrm{t}\mathrm{y}=\frac{\mathrm{T}\mathrm{r}\mathrm{u}\mathrm{e}\:\mathrm{N}\mathrm{e}\mathrm{g}\mathrm{a}\mathrm{t}\mathrm{i}\mathrm{v}\mathrm{e}\mathrm{s}}{\mathrm{T}\mathrm{r}\mathrm{u}\mathrm{e}\:\mathrm{N}\mathrm{e}\mathrm{g}\mathrm{a}\mathrm{t}\mathrm{i}\mathrm{v}\mathrm{e}\mathrm{s}+\mathrm{F}\mathrm{a}\mathrm{l}\mathrm{s}\mathrm{e}\:\mathrm{P}\mathrm{o}\mathrm{s}\mathrm{i}\mathrm{t}\mathrm{i}\mathrm{v}\mathrm{e}\mathrm{s}}$$
  It shows the ability to correctly identify negatives.
- False Discovery Rate (FDR).
  $$\:\mathrm{F}\mathrm{D}\mathrm{R}=\:\frac{\mathrm{F}\mathrm{a}\mathrm{l}\mathrm{s}\mathrm{e}\:\mathrm{P}\mathrm{o}\mathrm{s}\mathrm{i}\mathrm{t}\mathrm{i}\mathrm{v}\mathrm{e}\mathrm{s}}{\mathrm{T}\mathrm{r}\mathrm{u}\mathrm{e}\:\mathrm{P}\mathrm{o}\mathrm{s}\mathrm{i}\mathrm{t}\mathrm{i}\mathrm{v}\mathrm{e}\mathrm{s}+\mathrm{F}\mathrm{a}\mathrm{l}\mathrm{s}\mathrm{e}\:\mathrm{P}\mathrm{o}\mathrm{s}\mathrm{i}\mathrm{t}\mathrm{i}\mathrm{v}\mathrm{e}\mathrm{s}}=1-\mathrm{p}\mathrm{r}\mathrm{e}\mathrm{c}\mathrm{i}\mathrm{s}\mathrm{i}\mathrm{o}\mathrm{n}$$
- False Omission Rate (FOR).
  $$\:\mathrm{F}\mathrm{O}\mathrm{R}=\:\frac{\mathrm{F}\mathrm{a}\mathrm{l}\mathrm{s}\mathrm{e}\:\mathrm{N}\mathrm{e}\mathrm{g}\mathrm{a}\mathrm{t}\mathrm{i}\mathrm{v}\mathrm{e}\mathrm{s}}{\mathrm{T}\mathrm{r}\mathrm{u}\mathrm{e}\:\mathrm{N}\mathrm{e}\mathrm{g}\mathrm{a}\mathrm{t}\mathrm{i}\mathrm{v}\mathrm{e}\mathrm{s}+\mathrm{F}\mathrm{a}\mathrm{l}\mathrm{s}\mathrm{e}\:\mathrm{N}\mathrm{e}\mathrm{g}\mathrm{a}\mathrm{t}\mathrm{i}\mathrm{v}\mathrm{e}\mathrm{s}}=1-\mathrm{N}\mathrm{e}\mathrm{g}\mathrm{a}\mathrm{t}\mathrm{i}\mathrm{v}\mathrm{e}\:\mathrm{p}\mathrm{r}\mathrm{e}\mathrm{d}\mathrm{i}\mathrm{c}\mathrm{t}\mathrm{e}\mathrm{d}\:\mathrm{v}\mathrm{a}\mathrm{l}\mathrm{u}\mathrm{e}$$
  It shows the probability that a predicted negative is actually wrong.
- Cohen’s Kappa:
  $$\:\mathrm{k}=\:\frac{{\mathrm{p}}_{\mathrm{o}}-{\mathrm{p}}_{\mathrm{e}}}{1-{\mathrm{p}}_{\mathrm{e}}}$$
  $\:{p}_{0}$​: Observed Agreement $\:={p}_{0}=$​ Number of agreements​/Total number of samples.
  $\:{p}_{e}$​: Expected Agreement by Chance.
  it shows agreement between predictions and true labels, adjusted for chance.it is good for multi-class or imbalanced datasets.

### Explainability and robustness

Grad-CAM (Gradient-Weighted Activation Mapping) produces heatmaps that highlight important tumor regions.

25 $$\:{L}_{GradCAM}^{c}=ReLU\left(\sum\:_{k}{\alpha\:}_{k}^{c}{A}^{k}\right)$$

26 $$\:{\alpha\:}_{k}^{c}=\frac{1}{Z}\sum\:_{i}\sum\:_{j}\frac{\partial\:{y}^{c}}{\partial\:{A}_{ij}^{k}}$$

where.

$\:{A}^{k}$ are the feature maps of the final convolutional layer.

$\:{y}^{c}$ is the score for class c.

$\:{\alpha\:}_{k}^{c}$ is the importance weight of feature map k.

### Robustness analysis

Robustness was tested under two types of perturbations:

Additive gaussian noise:$\:\:{I}^{{\prime\:}}=I+N(0,\:{\sigma\:}^{2})$

Resolution Degradation: Downscaling to the certain percentages of the original image size, followed by bilinear upsampling. Accuracy under these degradations quantifies the stability of the model.

### SHAP (SHapley Additive exPlanations)

27 $$\:{\varphi\:}_{i}=\:{\sum\:}_{\left\{S\:\subseteq\:F\:\left\{i\right\}\right\}}\frac{\left|S\right|!\:,\:\left(\left|F\right|-\:\left|S\right|-\:1\right)!}{\left|F\right|!}\left(\left[f\right(S\:\cup\:\left\{i\right\}\right)-\:f\left(S\right)]$$

Where:

- F: all features.
- S: any subset of features not including i.
- f(S): model prediction using only features in S.
- ϕ_i_​: SHAP value → contribution of feature I to the model output, enabling interpretable, feature-level insights.

## Results and discussion

The proposed framework is modelled using the publicly available MRI scan dataset, the Kaggle MRI Brain tumor dataset^40^. This dataset represents brain tumors into fifteen classes, the normal brain tissue (NOR), astrocytoma (AST), carcinoma (CAR), ependymoma (EPE), ganglioglioma (GAN), germinoma (GER), glioblastoma (GLI), granuloma (GRA), medulloblastoma (MED), meningioma (MEN), neurocytoma (NEU), oligodendroglioma (OLI), papilloma (PAP), schwannoma (SCH), and tuberculoma (TUB). Although the original scans vary in dimensions, they are all formatted as JPG and are resized to a uniform resolution and grey images are converted into RGB images for processing within the proposed neural networks across different experimental conditions.

The original Kaggle dataset contains 4,456 MRI image samples. During preprocessing, we performed dataset-wide deduplication using MD5 hashing to remove identical scans. This procedure identified and removed 51 duplicate images, resulting in 4,405 unique MRI images used for experimentation. From the deduplicated set, patient IDs were programmatically extracted from filenames, and the dataset was partitioned strictly at the patient level (70%/15%/15%) so that all images from a given patient appear in only one split. The resulting splits contain 3,318/579/508 images, corresponding to 2,275/487/488 unique patients in the training, validation, and test sets, respectively, with zero patient overlap across splits. This design guarantees unbiased evaluation and robust generalization to unseen subjects (Table 1).

**Table 1:** MRI dataset distribution and patient-level split.

	Type of tumor	Raw MRI images	Images per patient-level split (Post-Dedup: 4,405)		
			Train	Val	Test
Radiologists’ classification	Astrocytoma	580	336	155	78
	Carcinoma	251	177	36	38
	Ependymoma	150	115	17	18
	Ganglioglioma	38	28	4	6
	Germinoma	100	80	8	12
	Glioblastoma	204	149	31	24
	Granuloma	78	61	6	11
	Medulloblastoma	131	90	16	25
	Meningioma	874	726	62	62
	Neurocytoma	457	378	40	39
	Normal	522	392	75	55
	Oligodendroglioma	224	150	32	27
	Papilloma	237	207	18	12
	Schwannoma	465	328	56	81
	Tuberculoma	145	101	23	20
	Raw dataset total (before deduplication)	4456	–	–	–
	Unique images used (after removing 51 duplicates)	4405	3318	579	508
	Unique patients per split		2275	487	488

### Dataset summary

The considered dataset has inherent class imbalance, ranging from 38 images for Ganglioglioma to 874 for Meningioma. To address this challenge and to enhance model robustness, a set of data augmentation, patient-level partitioning, and normalization procedures were adopted. The dataset was partitioned strictly at the patient level to prevent information leakage between training, validation, and testing subsets. After preprocessing and partitioning, the dataset contained 3,318 training images (2,275 unique patients), 579 validation images (487 unique patients), and 508 testing images (488 unique patients). Patient-level separation minimizes possible overfitting of the individual anatomical traits, thus increasing the model reliability in its use on external unseen clinical data. This strategy also avoids the potential data leakage that may occur when performing image-level k-fold cross-validation, where images from the same patient could appear in multiple folds.

The pre-processed dataset was resized to a fixed input dimension suitable for CNN-MS-DAM architecture, upholding spatial integrity and MRI modality features that are important in the process of correct classification. Data augmentation was applied only to the training set using techniques such as MixUp, geometric transformations, and intensity scaling to improve model generalization. These augmentations were performed online during training and did not generate permanently stored images; therefore, the dataset size remained unchanged. No augmentation was applied to the validation or test sets.

### Model training, validation and test performance

**Fig. 2:**
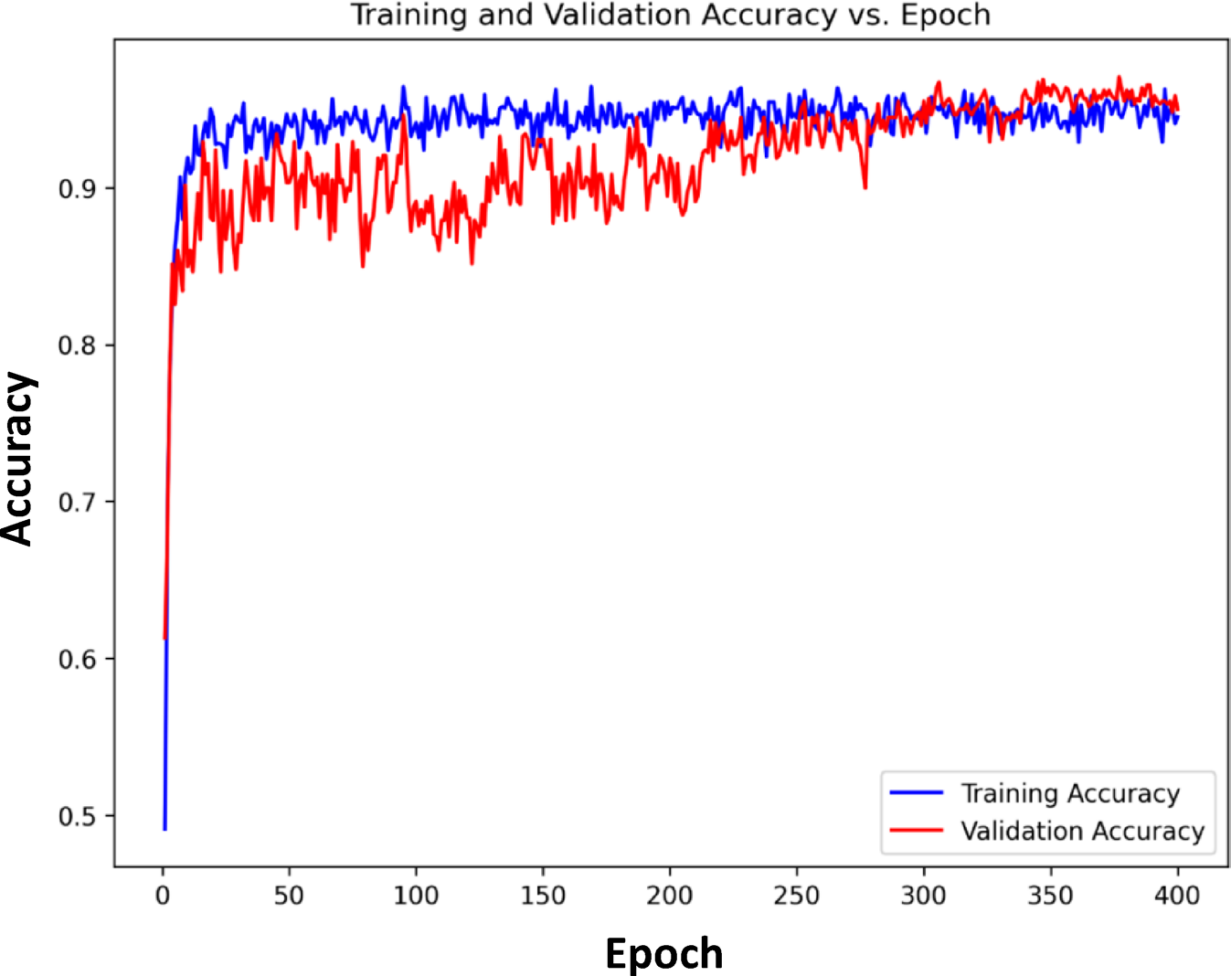
Convergence of training and validation accuracy with epochs.

**Fig. 3:**
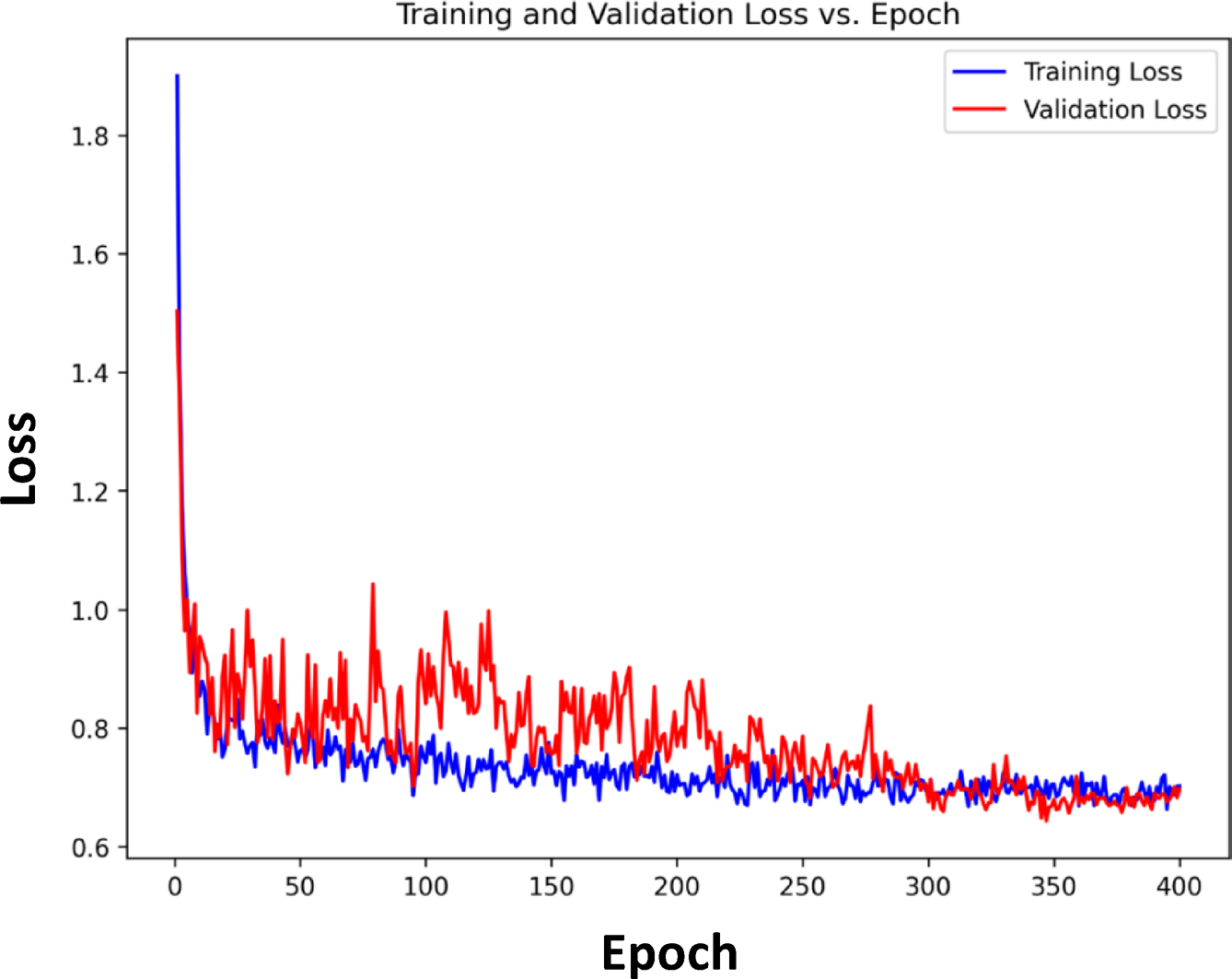
Convergence of training and validation loss with epochs.

Figures 2 and 3 demonstrate that the training and validation accuracies closely follow the corresponding loss curves, indicating stable convergence and minimal overfitting despite training for 400 epochs. Although the ResNet101 backbone was initialized with pretrained ImageNet weights, additional fine-tuning was necessary because MRI images differ significantly from natural images in texture and structural patterns. Moreover, the integration of the proposed MS-DAM module introduces additional learnable parameters that require extended optimization for proper convergence. Therefore, training was conducted for 400 epochs using a cosine annealing learning-rate scheduler, allowing gradual reduction of the learning rate and stable learning dynamics during later training stages. The consistent convergence behavior observed in Figs. 2 and 3 confirms that the model achieves stable optimization without overfitting. The detailed hyperparameter setting of the proposed MS-DAM-ResNet101 model is summarized in Table 2. All experiments were implemented in Python using the PyTorch framework and executed on the Kaggle platform using an NVIDIA Tesla P100 GPU accelerator (16 GB VRAM). Strong data augmentations, such as Gaussian noise, random intensity scaling, perspective transformations and MixUp regularisation, enhanced the robustness of the model and avoided memorization of the training set. The original dataset employed in this study contains 4,405 unique MRI images. Data augmentation was applied online through the training process only, where augmented samples are created dynamically at each training iteration rather than being permanently stored in the dataset. So, the number of images stored before and after augmentation remains 4,405 images. No augmentation was applied to the validation or test sets in order to assure an unbiased evaluation of model performance. The dataset was divided into training, validation, and testing subsets. The validation set was used only to monitor training performance and select the best-performing model during training, while the final performance evaluation and comparisons were conducted exclusively on the independent test set, which remained completely unseen during model development.

Multi-scale contextual dependencies and spatial variations among tumour regions were reported in the MS-DAM module which resulted in more discriminative feature representations. Optimal validation performance of 96.89% and a loss of 0.6435 were observed at epoch 347; after that the performance remained stable with slight variations indicating the convergence of the optimiser and the feature representation. In subsequent epochs, minor fluctuations can be explained by the dynamic interactions between the deformable attention offsets and balancing at the level of classes when there is the fusion of fine-grained features. Further the best-loss model is tested using the test dataset indicated that there is strong generalization, where a test accuracy at 99.21% and a test loss at 0.1545 were obtained. To further assess the quality of the learned feature representations, an SVM classifier was trained using the penultimate-layer embeddings of the proposed network. The SVM achieved a validation accuracy of 97.06%, indicating that the extracted features are highly discriminative and well-structured even when evaluated with a classical classifier.

**Table 2:** Hyperparameter configuration of the proposed MS-DAM–ResNet101 model.

Category	Parameter	Value
General settings	Random seed	42
	Batch size	16
	Input size	224 × 224
	Epochs	400
Optimizer	Optimizer type	AdamW
	Initial learning rate	1 × 10⁻⁴
	Weight decay	1 × 10⁻⁴
	LR scheduler	Cosine annealing
	T_max	400
	η_min	1 × 10⁻⁶
Loss function	Cross-entropy	With label smoothing (0.1)
Regularization	Dropout	0.4
	MixUp	α = 0.1
MS-DAM module	Input channels	[256, 512, 1024, 2048]
	Output channels	512
	Offset scale	0.12
	Spatial attention	Enabled
	Channel attention	SE block enabled
Data augmentation	Random crop scale	0.85–1.0
	Horizontal flip	*p* = 0.5
	Vertical flip	*p* = 0.3
	Rotation	± 5°
	Gaussian noise	std = 0.005
	Random erasing	*p* = 0.1
Data split	Training	70%
	Validation	15%
	Testing	15%
	Split type	Patient-wise

This result confirms that the strong performance of the proposed model primarily stems from the robustness and separability of the learned feature representations rather than from the final fully connected classification layer. Overall, the training curves and evaluation process prove that the MS-DAM-based ResNet architecture is well converged, highly generalized and provides superior classification results on multi-class brain-tumour MRI images. Although the validation accuracy (96.89%) is slightly lower than the test accuracy (99.21%), this difference can arise from natural variability introduced by random patient-level splitting and class distribution differences across subsets. Importantly, no hyperparameters were tuned using the test set. The test data remained strictly held out until final evaluation.

In order to examine the computational efficiency of the proposed architecture, Table 3 summarizes the computational features of the proposed model together with representative backbone architectures. The comparison includes the number of trainable parameters, GFLOPs, inference time per image, and the overall time taken during training models. The proposed MS-DAM-ResNet101 model contains approximately 56.93 million trainable parameters and requires 47.50 GFLOPs, reflecting the additional computational cost introduced by the multi-scale deformable attention module. Although the model capacity is increased, the inference latency remains low (7.46 ms per image), which means that the model can facilitate real-time prediction across most of the situations. The overall time of training on the proposed model was around 9 h and 23 min on the specified GPU platform. No automated hyperparameter optimization techniques were employed; therefore, the reported computational time reflects only the cost of model training and validation.

### Confusion matrix analysis

**Fig. 4:**
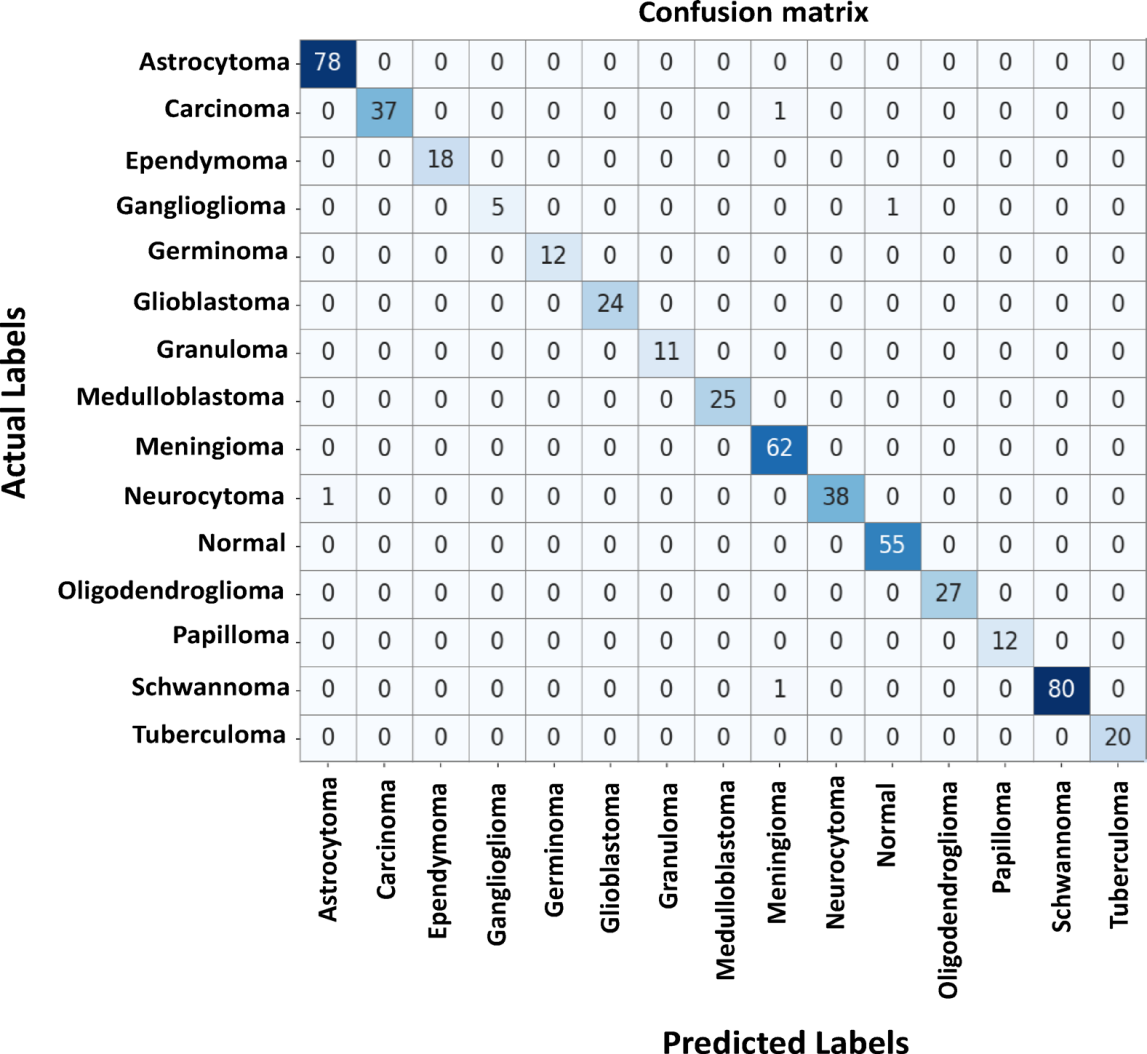
Confusion heatmap of predicted vs. actual tumor classes.

Figure 4 shows the confusion matrix with the incorrectly classified counts and the corresponding normalized percentages for all types of brain tumors. The proposed ResNet101 with Multi-Scale Deformable Attention Module (MS-DAM) demonstrated outstanding per-class recognition with very few misclassifications. The diagonal dominance confirms the fact that the majority samples were correctly classified, as displayed in both the incorrectly classified count and normalized percentage values, reflecting the model’s high confidence and robust feature learning across different types of tumors. As an example, Astrocytoma (78 cases, 100%), Ependymoma (18 cases, 100%), Germinoma (12 cases, 100%), Glioblastoma (24 cases, 100%), Granuloma (11 samples, 100%), Medulloblastoma (25 cases, 100%), Meningioma (62 samples, 100%), Normal (55 samples, 100%), Oligodendroglioma (27 samples, 100%), Papilloma (12 samples, 100%) and Tuberculoma (20 samples, 100%) were classified perfectly.

Slight confusion showed in Schwannoma, 80 predictions being correct (98.8%) and only one being wrongly classified (1.2%) as Meningioma, they may be because of similarity in the texture patterns. Neurocytoma shown 38 correct (97.4%) and only one misclassification as Astrocytoma, Carcinoma 37 predictions being correct (97.4%) and only one being wrongly classified (2.6%) as Meningioma, while Ganglioglioma had 5 correct (83.3%) with only one being misclassified (16.7%) as Normal. Overall, the matrix’s strong diagonal intensity in incorrectly classified count. This indicates that the proposed ResNet101 based on MS-DAM effectively learns class-specific spatial and contextual features, resulting in balanced classification performance and superior generalization of all brain tumor types.

### Quantitative evaluation metrics

The classification results shown in Table 4 indicate an overall strong performance of the proposed model in comparison with the Zarenia et al.^4^, while improvements vary depending on the tumor class and metric. With regards to Accuracy, the present results are showing consistently high values with a range of between 0.996 and 1.000 in all classes, which indicates reliable correct prediction rates. There is only one class i.e., Meningioma, 0.969 with slightly lower precision than Zarenia et al.^4^ values (0.977), and the remaining all other types of tumors are perfect at 1.000 which is the same or even higher than the performance of the Zarenia et al.^4^. The metrics aggregation Precision, Recall, F1 -score, Dice coefficient, Specificity, and Cohen Kappa serve as indicators of almost perfect agreement between the ground-truth labels and the model on most tumor types. A detailed class by class comparison reveals that there are many categories of tumors that show improved or the identical performance as in the Zarenia et al.^4^ model. Specifically, Ependymoma, Germinoma, Glioblastoma, Medulloblastoma, Oligodendroglioma, Papilloma, and Tuberculoma have a perfect Precision and Recall (1.000), at the same level or better than the one obtained by Zarenia et al.^4^. Likewise, Astrocytoma, Carcinoma, Normal tissue and Schwannoma have very high Precision $\:(\ge\:\:0.982)$ with high Recall $\:(\ge\:0.974)$, giving rise to F1 and Dice close to 0.99. Macro-Level Agreement is also very high, with Cohen’s Kappa values close to unity for several classes, indicating the high reliability of the model across the dataset.

Although the performance is overall good, there is some minor decline in a few classes when compared to the Zarenia et al.^4^ method. The greatest drop is in Ganglioglioma, with Recall dropping from 1.000 to 0.833. This drop makes the F1-score drop to approximately 0.909. This drop can be explained by less supporting classes or higher intra-class variability that makes the generalization hard. This can be alleviated by targeted data augmentation, resampling or detailed analysis of samples that were misclassified.

Carcinoma and Schwannoma also show small decreases - Recall decreases from 0.979 to 0.974 and from 0.989 to 0.988 respectively. These changes are very small and have no significant impact on the clinical usability of the model. Importantly, the more challenging categories - e.g. Granuloma and Ependymoma - display improved or stable Precision and Recall with the current model. This implies less false positive and false negative for these categories. Error rate measures such as False Discovery Rate (FDR) and False Negative Rate (FNR) remain very low across all classes (FDR ≈ 0.000–0.034; FNR $\:\le\:0.166$ for the worst case) and specificity values are uniformly high $\:(\approx\:\:0.996\:-\:1.000).$ The Present method significantly controls the false FDR and the FNR significantly compared with the Zarenia et al.^4^. Most classes are 0.000 for FDR and FNR. In contrast, the values for the Zarenia et al.^4^ model are higher for FDR values (up to 0.125) and also significant FNR values, which indicate a weaker sensitivity and specificity. In total, the existing model provides clinically reliable, stable, and high-accurate performance in most tumor types. Although Zarenia et al.^4^ excel in some of the categories, they report metrics in fewer detail and their accuracy and recall vary more.

### Performance evaluation of Precision–Recall, Receiver Operating Characteristic (ROC) curve and AUC & t-SNE feature visualization

**Fig. 5:**
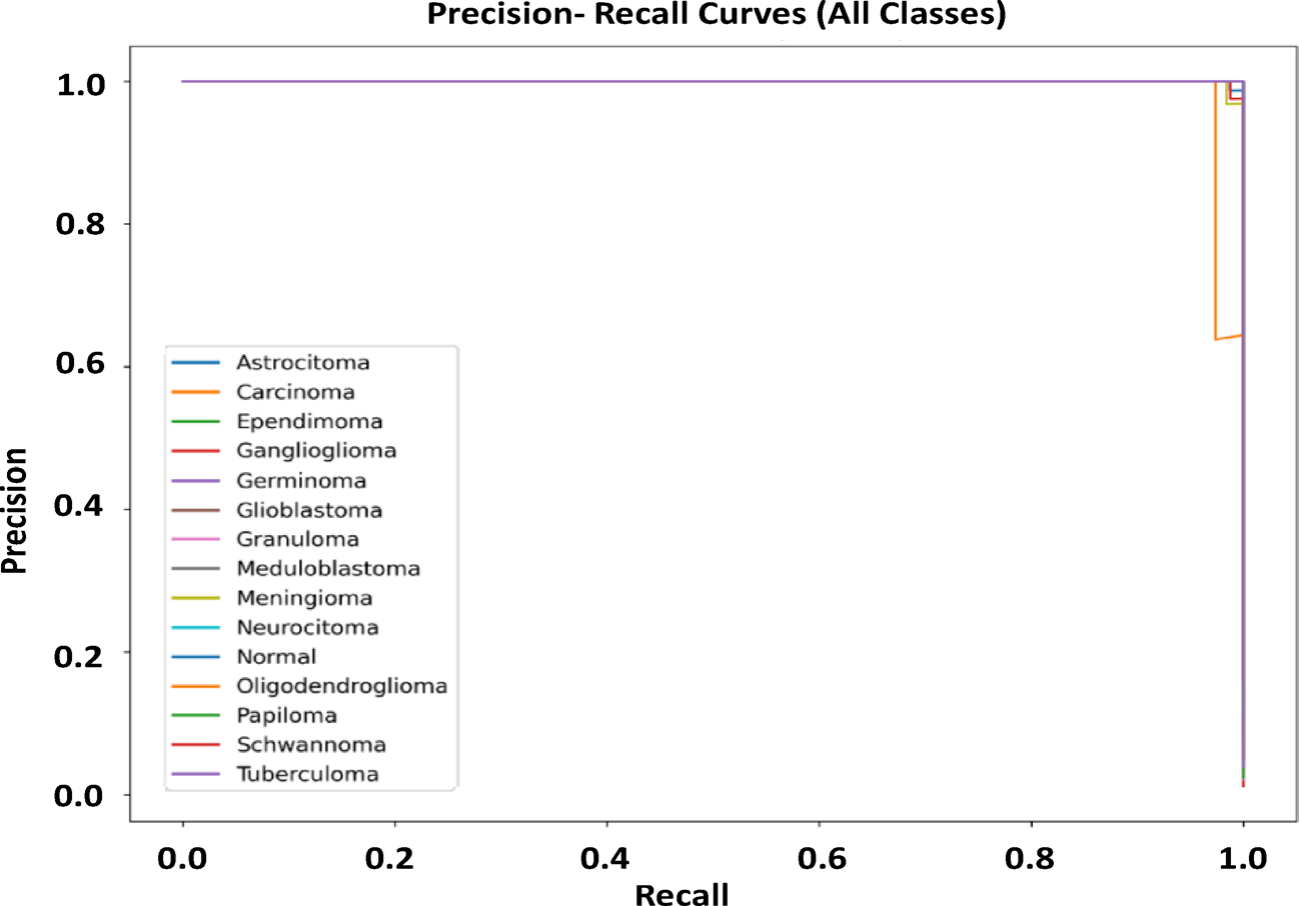
Precision–Recall curves for all brain tumor classes using the proposed model.

**Fig. 6:**
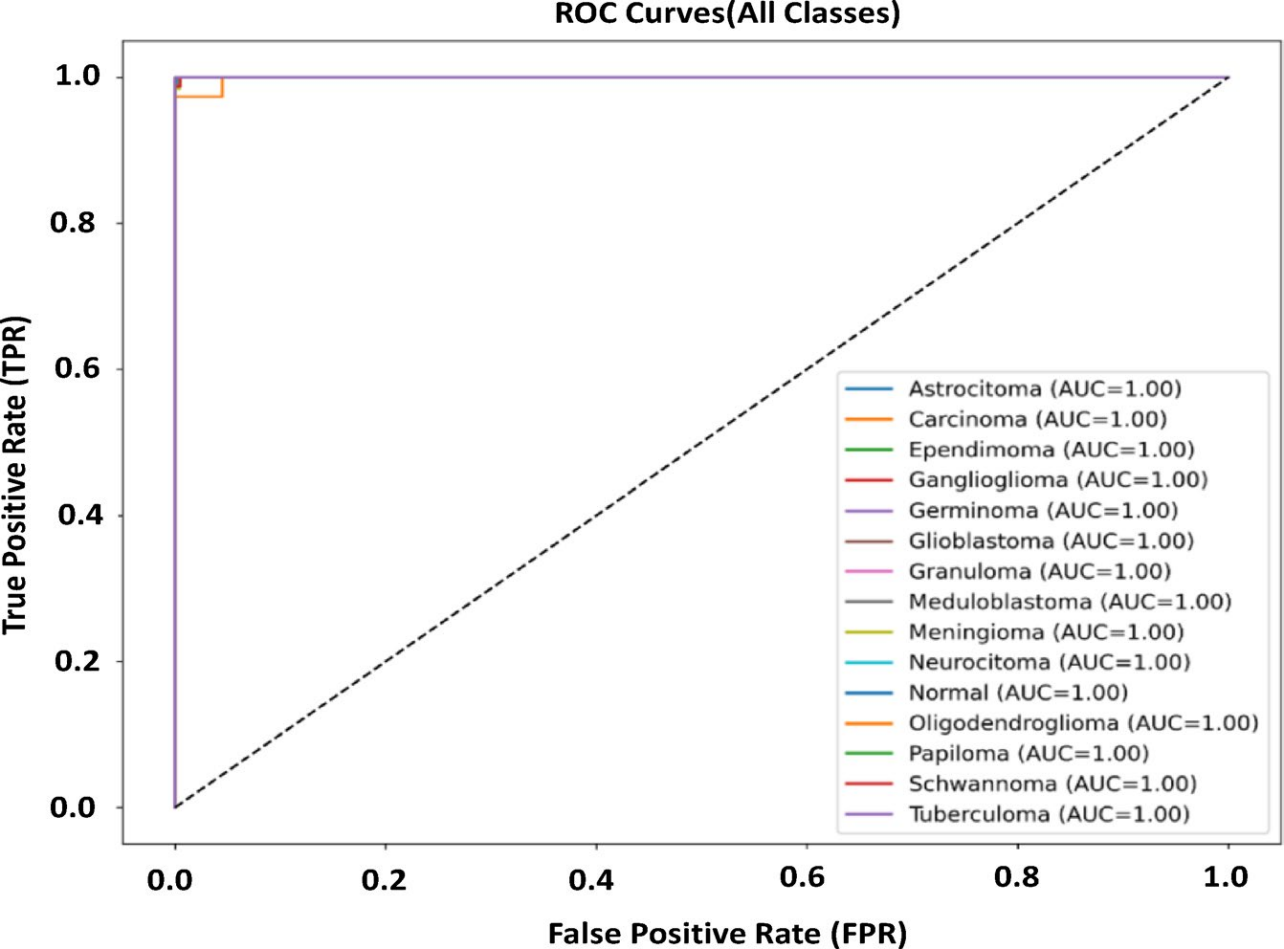
Receiver Operating Characteristic (ROC) curves for all brain tumor classes using the proposed model.

**Fig. 7:**
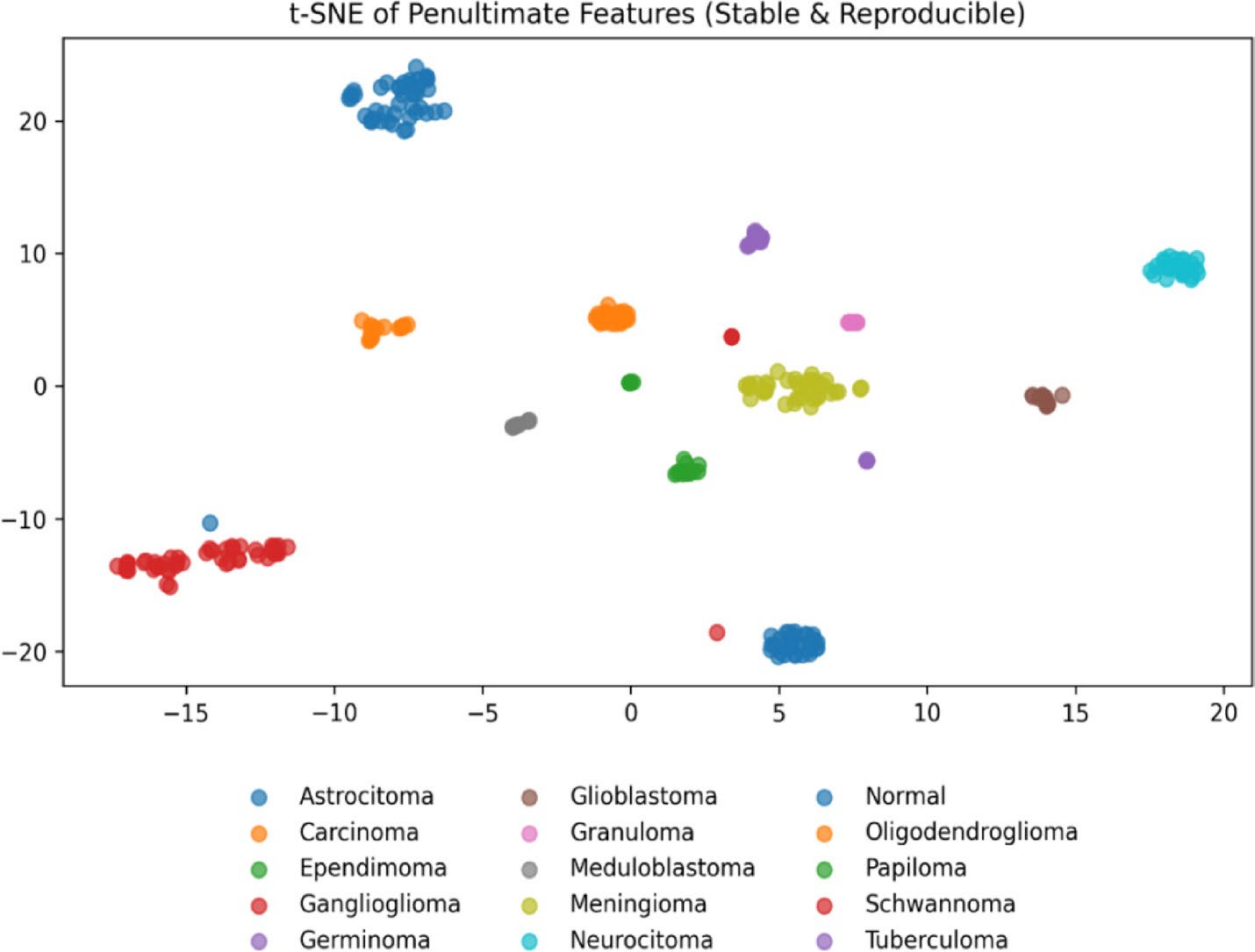
t-SNE visualization of the penultimate feature representations extracted by the proposed CNN–MS-DAM model for multiclass brain tumor classification.

Figures 5, 6 and 7 present ROC, Precision-Recall (PR) and t-SNE results of the proposed multiclass brain tumor classifier. These results collectively demonstrate the strong discriminative power, reliability, and distinct features separation of the model. As seen in the PR curves, the model is very precise at all recall levels, indicating that it is robust to class imbalance and, at the same time, keeps the false-positive rates low at even in less represented tumor types.

All types of tumors are separable near-perfectly by the ROC curves with AUCs of 1.0. All curves are near the top-left area which means that there is a very high sensitivity and specificity across all categories. These findings are supported by the t -SNE visualization, which depict distinct, tight clusters of each tumor type in the latent space. Class overlap is minimal, an indication that the model reflects unique, and discriminative characteristics of MRI-based scans. While some classes achieved AUC values of 1.00, this reflects near-complete separability within this dataset. However, we acknowledge that independent external validation across institutions would further strengthen generalizability claims.

**Table 3:** Computational characteristics of the proposed model and representative baseline architectures.

Model	Trainable parameters (million)	GFLOPs	Inference time (ms per image)	Training time
EfficientNet-B0 (baseline reference)	7.25 M	10.15	2.5	3 h 43 m
ResNet50 (baseline reference)	37.94 M	43.77	6.43	8 h 2 m
ResNet101 + MS-DAM (proposed model)	56.93 M	47.50	7.46	9 h 23 m

**Table 4:** Class-wise performance comparison of the proposed model with Ref^4^.

Class	Method	Accuracy	Precision	Recall	F1	Dice	Specificity	FDR	FOR	FNR	Kappa
AST	Ref^4^	–	0.966	0.957	–	–	–	0.034	–	0.043	–
	Present	0.998	0.987	1.000	0.994	0.994	0.998	0.013	0.000	0.000	0.992
CAR	Ref^4^	–	0.940	0.979	–	–	–	0.060	–	0.021	–
	Present	0.998	1.000	0.974	0.987	0.987	1.000	0.000	0.002	0.026	0.986
EPE	Ref^4^	–	1.000	1.000	–	–	–	0.000	–	0.000	–
	Present	1.000	1.000	1.000	1.000	1.000	1.000	0.000	0.000	0.000	1.000
GAN	Ref^4^	–	0.875	1.000	–	–	–	0.125	–	0.000	–
	Present	0.998	1.000	0.833	0.909	0.909	1.000	0.000	0.002	0.166	0.908
GER	Ref^4^	–	0.950	1.000	–	–	–	0.050	–	0.000	–
	Present	1.000	1.000	1.000	1.000	1.000	1.000	0.000	0.000	0.000	1.000
GLI	Ref^4^	–	0.976	1.000	–	–	–	0.024	–	0.000	–
	Present	1.000	1.000	1.000	1.000	1.000	1.000	0.000	0.000	0.000	1.000
GRA	Ref^4^	–	0.875	0.875	–	–	–	0.125	–	0.125	–
	Present	1.000	1.000	1.000	1.000	1.000	1.000	0.000	0.000	0.000	1.000
MED	Ref^4^	–	0.962	1.000	–	–	–	0.038	–	0.000	–
	Present	1.000	1.000	1.000	1.000	1.000	1.000	0.000	0.000	0.000	1.000
MEN	Ref^4^	–	0.977	0.955	–	–	–	0.023	–	0.045	–
	Present	0.996	0.969	1.000	0.984	0.984	0.996	0.031	0.000	0.000	0.982
NEU	Ref^4^	–	0.967	0.967	–	–	–	0.033	–	0.033	–
	Present	0.998	1.000	0.974	0.987	0.987	1.000	0.000	0.002	0.025	0.986
NOR	Ref^4^	–	0.962	0.962	–	–	–	0.038	–	0.038	–
	Present	0.998	0.982	1.000	0.991	0.991	0.998	0.018	0.000	0.000	0.990
OLI	Ref^4^	–	1.000	0.978	–	–	–	0.000	–	0.022	–
	Present	1.000	1.000	1.000	1.000	1.000	1.000	0.000	0.000	0.000	1.000
PAP	Ref^4^	–	0.979	0.958	–	–	–	0.021	–	0.042	–
	Present	1.000	1.000	1.000	1.000	1.000	1.000	0.000	0.000	0.000	1.000
SCH	Ref^4^	–	0.989	0.989	–	–	–	0.011	–	0.011	–
	Present	0.998	1.000	0.988	0.994	0.994	1.000	0.000	0.002	0.012	0.993
TUB	Ref^4^	–	0.931	0.964	–	–	–	0.069	–	–	
	Present	1.000	1.000	1.000	1.000	1.000	1.000	0.000	0.000	0.000	1.000
Macro Avg	Present	0.999	0.996	0.985	0.99	0.99	0.999	0.004	0.000	0.015	0.99
Micro Avg	Present	0.984	0.992	0.992	0.99	0.99	0	0.008	0	0.008	0.99

**Fig. 8:**
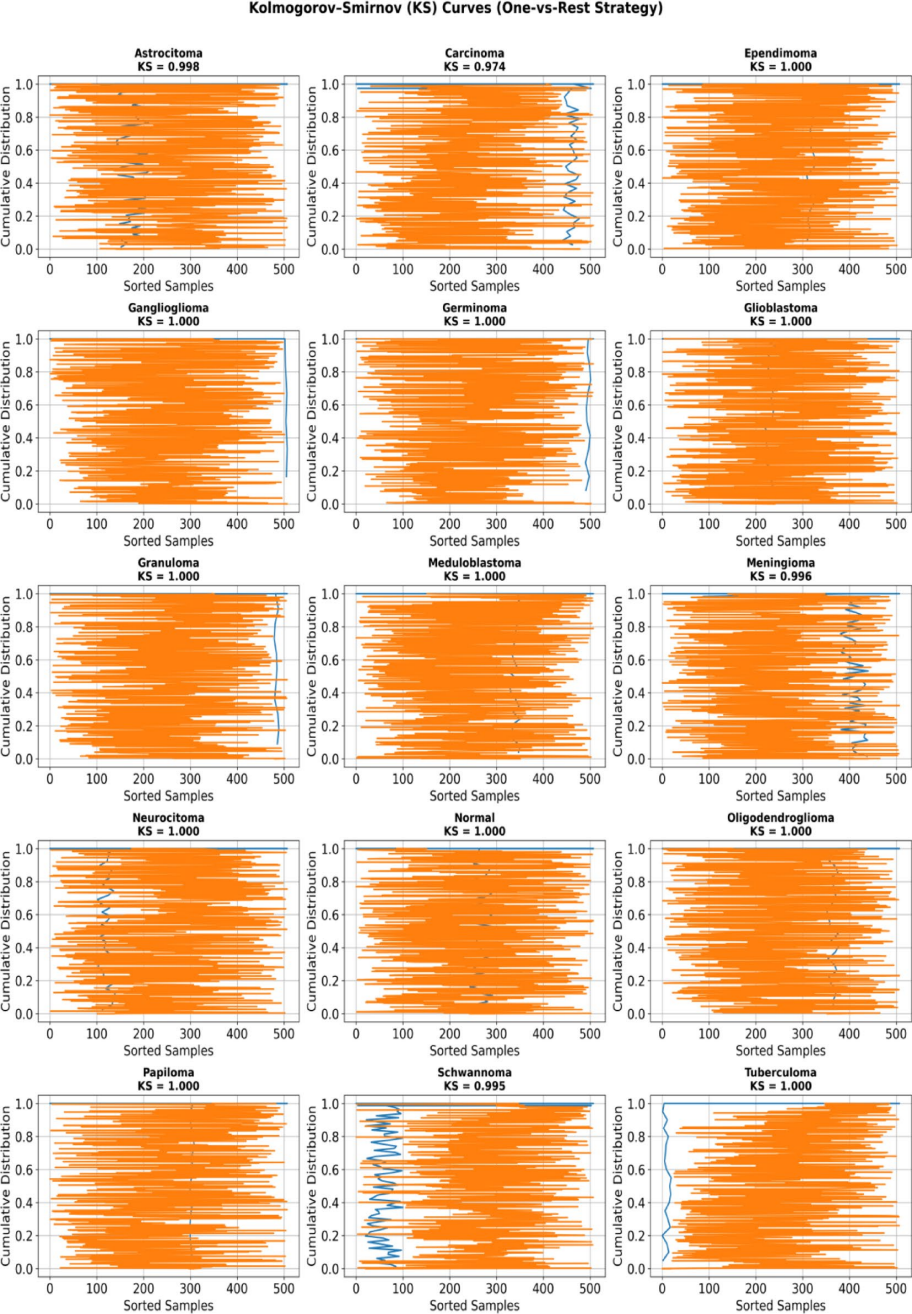
KS curves for all tumor classes using one-vs-rest strategy.

To further evaluate the discriminative capability of the proposed model, Kolmogorov–Smirnov (KS) analysis was performed using a one-vs-rest strategy across all 15 tumor categories and shown in Fig. 8. The KS statistic measures the maximum separation between cumulative positive and negative distributions, indicating the model’s ability to distinguish each class from the remaining categories. The overall Kolmogorov–Smirnov (KS) analysis revealed an average KS value of 0.9975, with a maximum KS of 1.0000 and a minimum KS of 0.9737, indicating exceptionally strong and consistent class separability across all tumor categories.

### Analysis of misclassified samples

**Fig. 9:**
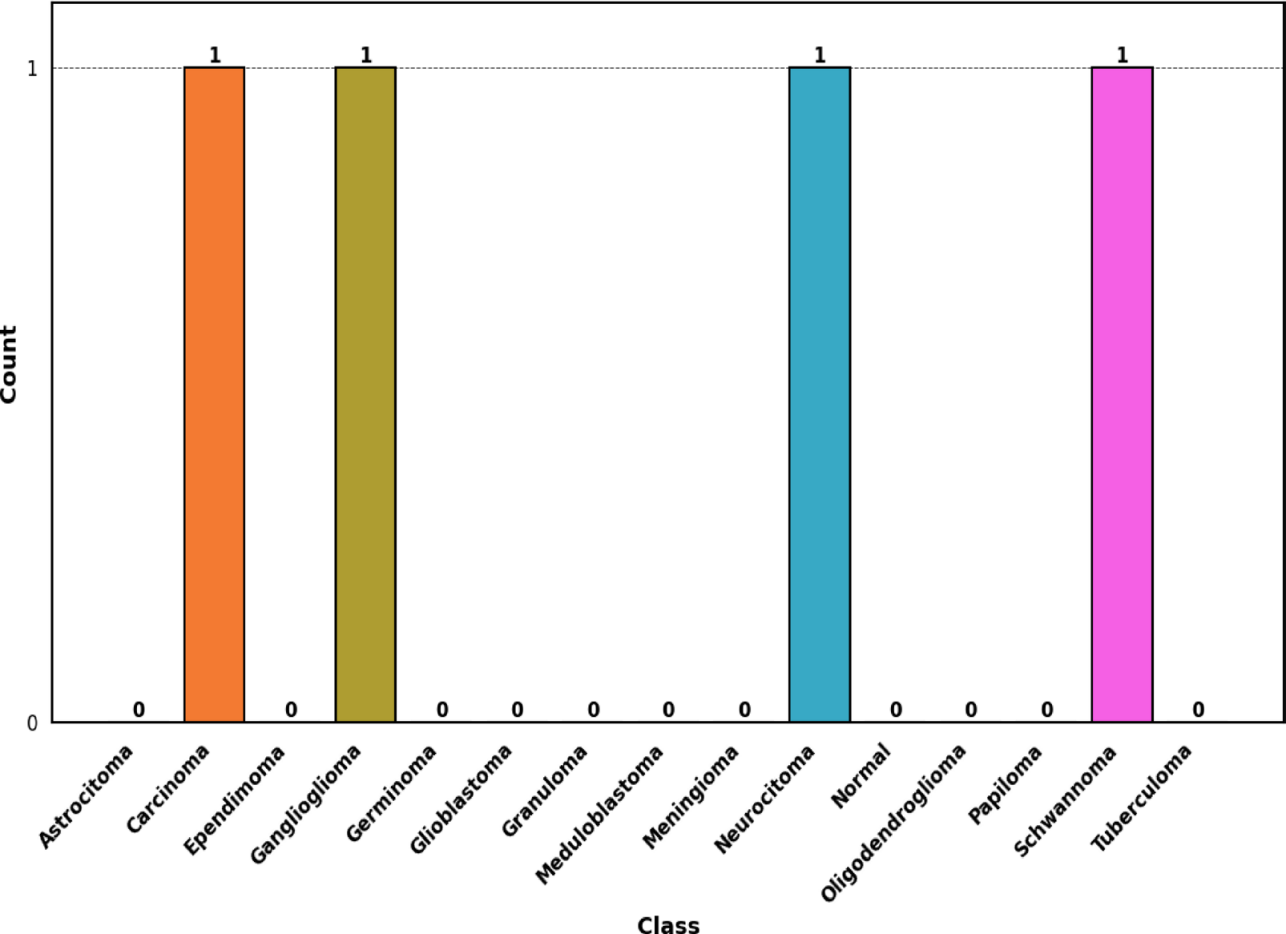
Representative misclassified MRI samples.

The representative incorrectly classified samples count per class is shown in Fig. 9. The misclassification distribution has shown that the CNN–MS–DAM model is quite stable on a per–class level, most types of tumor have zero or few errors. Carcinoma, Ganglioglioma, Neurocytoma and Schwannoma were the only misclassified 1 sample each and this is likely due to their overlapping MRI signal characteristics with other tumor types. Carcinomas and Gangliogliomas often exhibit nonspecific patterns that resemble other gliomas, while Neurocytomas lose their key location-based cues after ROI cropping, making them visually similar to ependymoma-like lesions. Schwannomas also share enhancement and boundary features with meningiomas, leading to confusion between these benign extra-axial tumors. These findings show that the model is able to learn discriminative, invariant characteristics in different tumor morphologies. The small number of, clinically explicable, incorrect classifications ensure solid generalization, high attention -based localization and localized decision limits.

### Classification performance for test data samples

**Fig. 10:**
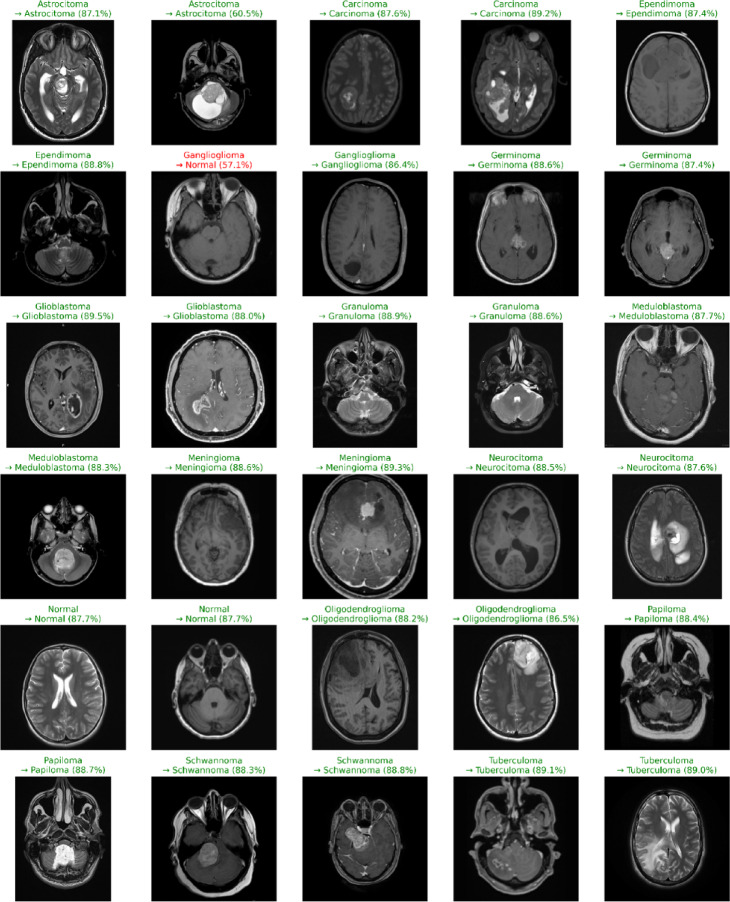
Classification results of brain MRI test images using the proposed algorithm.

The results of the proposed brain tumor classification algorithm on a sample of representative test MRI scans are shown in Fig. 10. The ground-truth, the label prediction by the model, as well as the confidence score are displayed in each scan corresponds to a distinct patient. The correctly classified examples are indicated by green, whereas only one misclassification example in red (a Ganglioglioma predicted as Normal) out of 30 samples drawn, which is a clear demonstration of the strong discriminative performance of the model. The proposed algorithm exhibits high classification accuracy with a wide range of tumor types of Astrocytoma, Ependymoma, Glioblastoma, Meningioma, Oligodendroglioma, Medulloblastoma, Schwannoma, Germinoma, Papiloma, Tuberculoma and a Normal tissue demonstrating its capability to capture subtle variations in morphology, intensity distribution, and regional contrast. Most predictions exhibit high confidence $\:(>87-90\%),$ indicating stable latent-space separation and reliable feature discrimination.

Misclassification (red) mostly occurs in class with similar anatomical or textural related classes. These incorrect classifications are associated with overlapping features of MRI images, tumor boundary ambiguity, or a small size of such cases in the training set. However, the algorithm is stable to different pathologies and imaging protocols. The high generalization ability demonstrated here confirms the effectiveness of the hybrid CNN based architecture (e.g., CNN -SVM, ResNet-ensemble, etc.) in differentiating the tumor subtypes. These accurate predictions on the heterogeneous MRI scans validate that the layers of feature-extraction acquire representations that have some sense, and that the classifier transforms them to the correct categories. The test accuracy and test loss of the proposed model were 99.21% and 0.1545 respectively in 508 samples. The average test loss of 0.1545 indicates that the model is stable across different batches and does not exhibit a noteworthy overfitting or performance deterioration.

### Robustness evaluation using bootstrap resampling

**Fig. 11:**
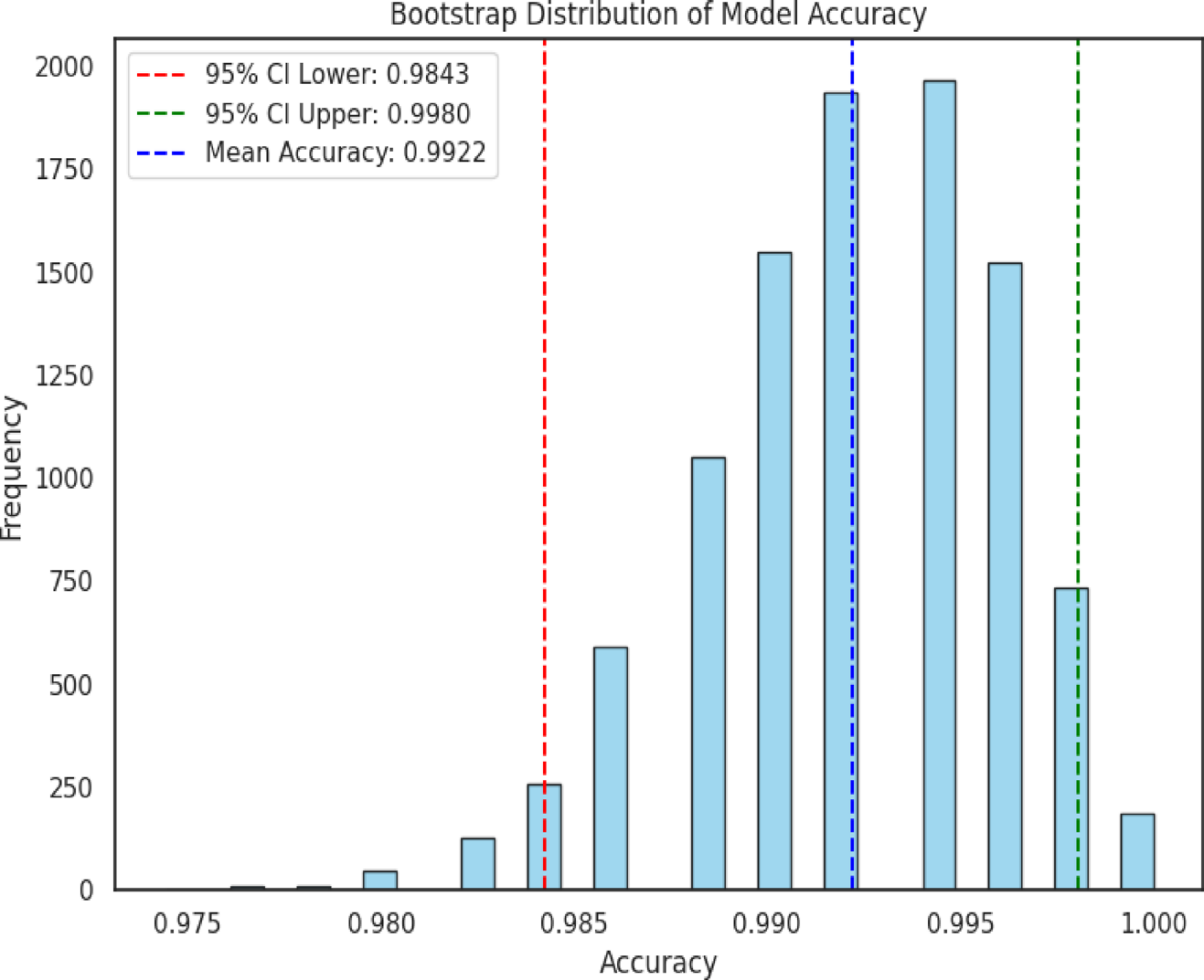
Bootstrap distribution of model accuracy for the multiclass brain tumor classification task.

The multiclass brain tumor classification model achieved a bootstrapped mean accuracy of test predictions yielded $\:99.21\:\pm\:\:0.40\%$ (95% confidence Interval: [0.9843, 0.9980]) validating the exceptional consistency and reliability with 10,000 resampled datasets. These statistics show that the model can be both highly accurate and robust in dissimilar data partitions. The low standard error of the mean (negligible, about 0.00394) also supports the accuracy of the estimated precision. Altogether, the findings suggest that the model can reliably distinguish between different types of tumors with minimal variability, suggesting strong generalization potential and stability for clinical or research applications. The stability and robustness of the performance of the model would be visualised in a histogram (Fig. 11) with the annotated accuracy of the 95% confidence range.

### Robustness evaluation

**Fig. 12:**
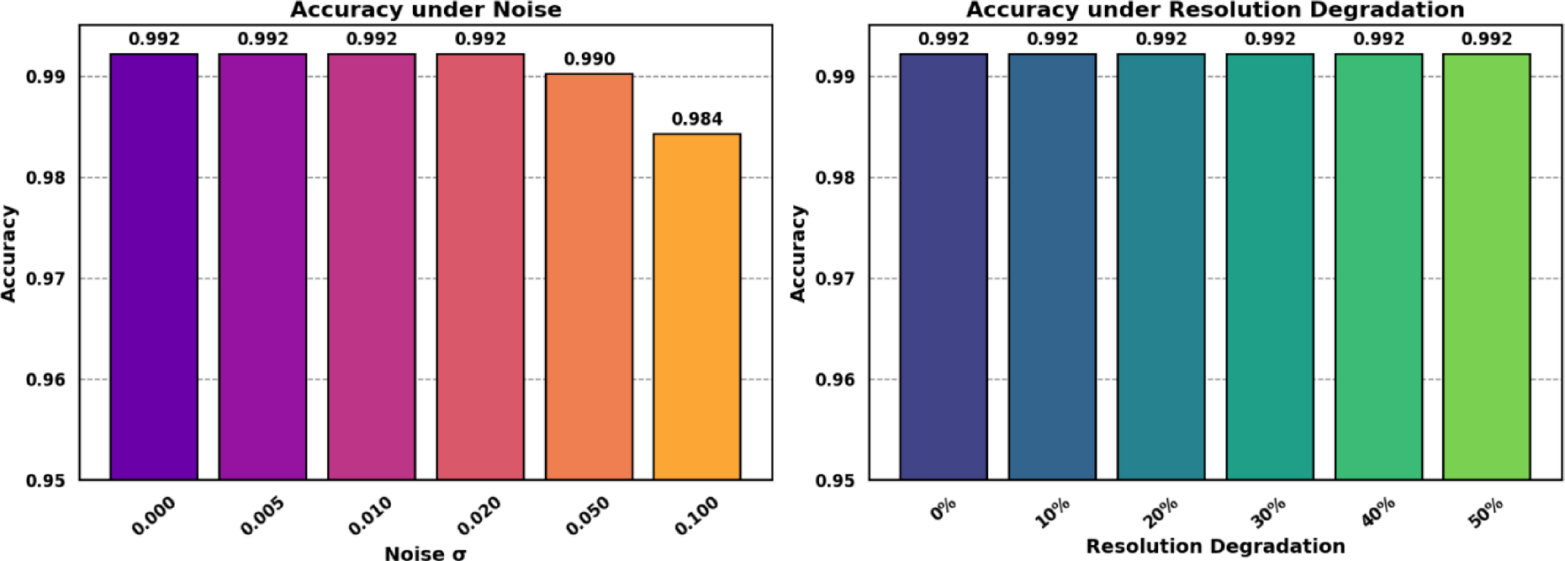
Effect of Gaussian noise and resolution degradation on classification accuracy.

Figure 12 left bar plot depicts the influence of the increase of Gaussian noise levels on the accuracy of the proposed model. The range of the noise level is between $\:\sigma\:=\:0$ (no noise) and 0.1. The accuracy at all noise levels 0, 0.005, 0.01 is quite stable with 99.21. There is a slight decrease in accuracy at the most noise level (σ = 0.05, and 0.1), with the value of 99.0 and 98.4%. This can denote the model’s robustness to slight noise or a potential regularization effect introduced by noise augmentation. The right bar plot in Fig. 12 shows the effect of decrease of Resolution of images on model accuracy. The accuracy remains at 99.21% up to 50% decrease. The proposed model exhibits robustness to noise, as well as moderate resolution loss, which is essential in medical imaging in the real world when the image quality of an image can fluctuate.

### Qualitative analysis MRI samples through Grad-CAM visualization

**Fig. 13:**
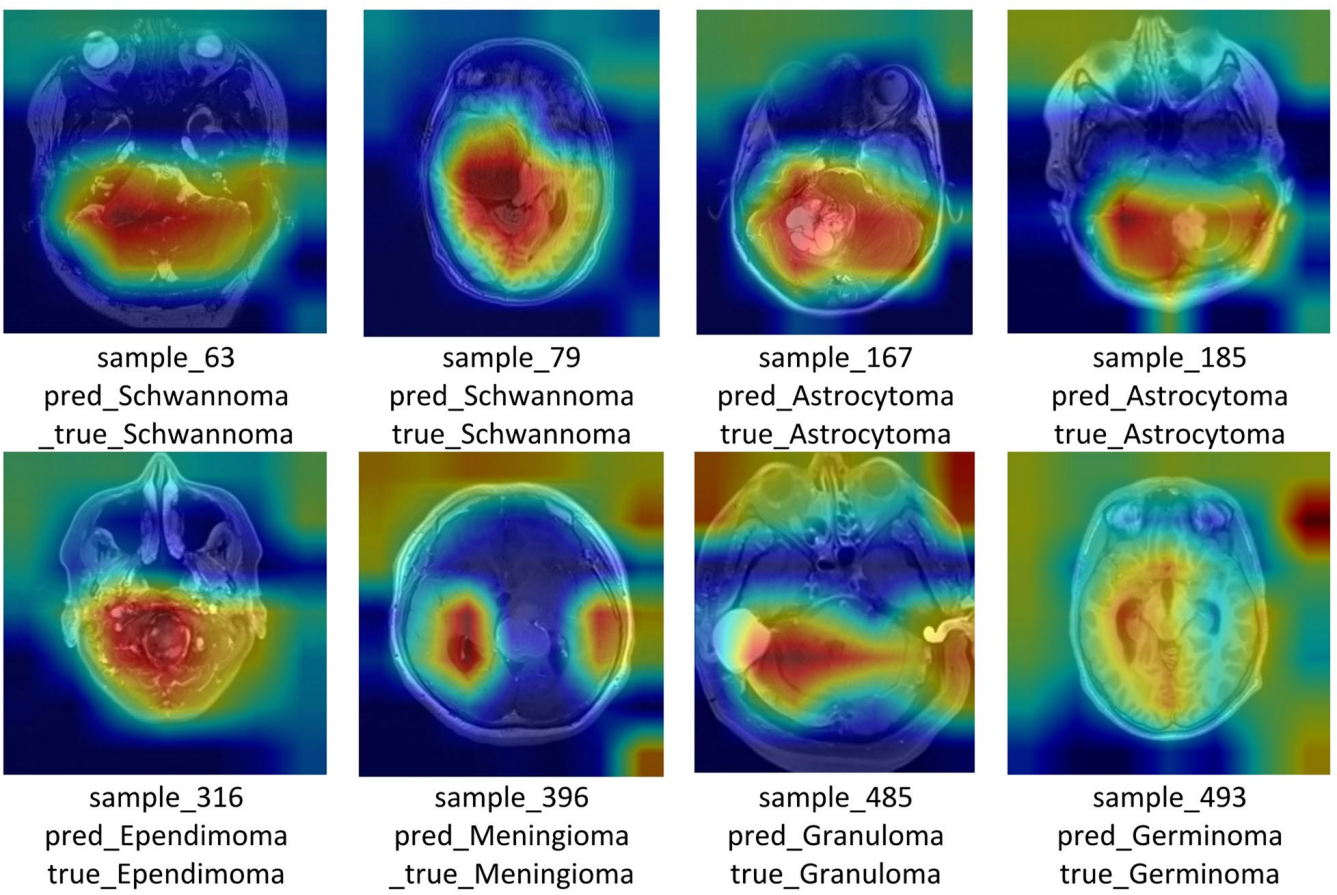
Representative Grad-CAM visualizations for correctly classified MRI brain tumor samples.

The Grad-CAM visualizations for correctly classified samples of MRI brain turmor are shown in Fig. 13. The Fig. provides the qualitative insights into the decision-making process of the proposed ResNet-MS-DAM deep learning model. The red and yellow highlighted activation regions indicate areas that most strongly influenced each prediction. The heatmaps of Schwannoma and Astrocytoma samples show concentrated activations within the tumor boundaries, indicating accurate localization of discriminative structural and intensity features. The Ependymoma and Meningioma maps indicate a specific focal emphasis of the ventricular and extra-axial areas, respectively, in accordance with anatomical characteristics. Similarly, the cases of Granuloma and Germinoma demonstrate a precise attention on the areas of focal lesions, which highlights the ability of the model to generalize across several tumor types.

### SHAP based explainability analysis

**Fig. 14:**
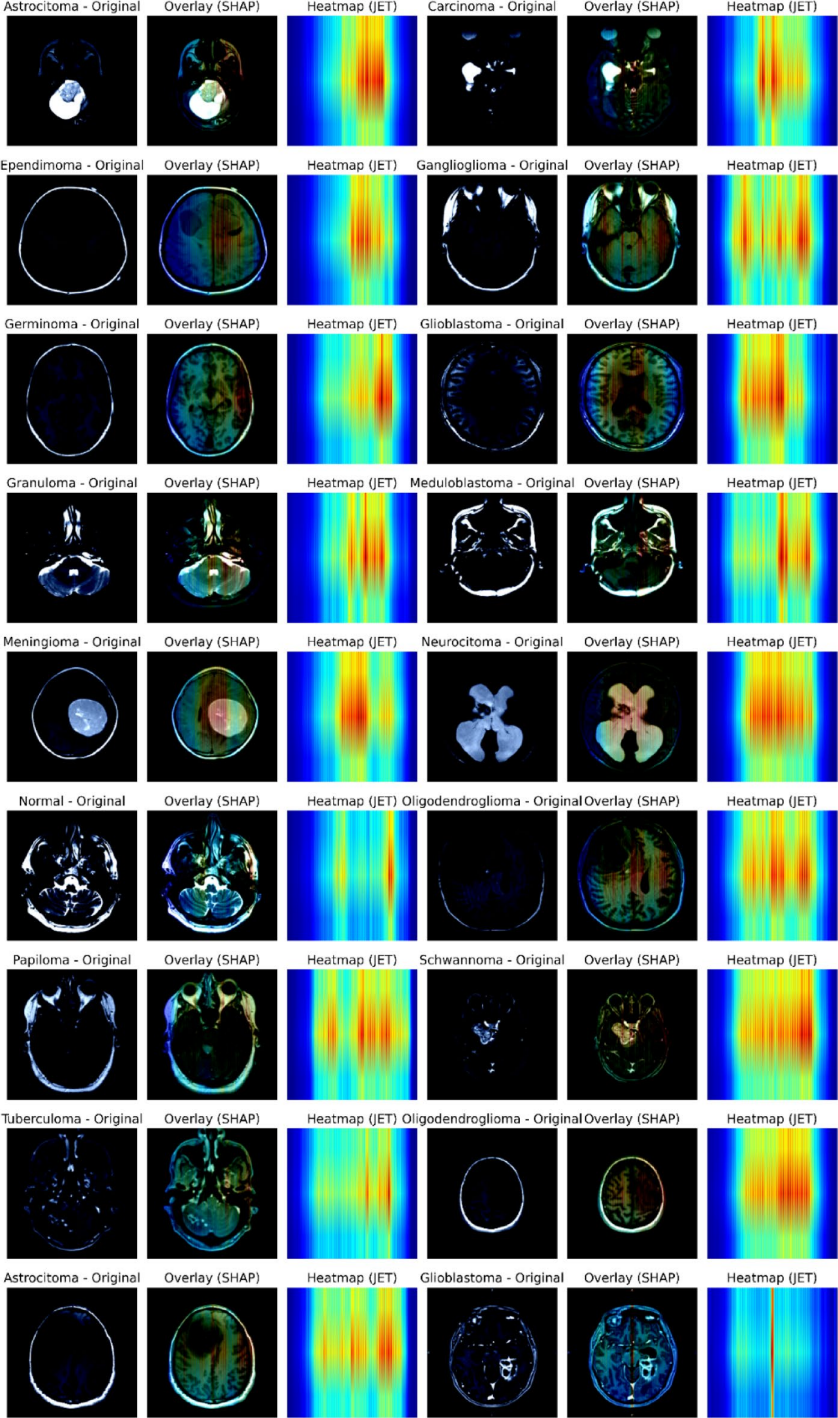
Representative SHAP visualizations for different brain tumor classes.

In order to decipher the predictions of the model and to evaluate the attribute of features, we conducted SHAP (SHapley Additive exPlanations) analysis on representative samples of MRI of various types of brain tumors (Fig. 14). SHAP offers the interpretability at pixel level by quantifying the contribution of each region to the output of a model. The overlaid SHAP maps indicate that the regions have a positive influence (red/yellow) or negative influence (blue) on the classification decision.

The SHAP visualizations of all tumor types show that the model is primarily concerned with tumor-affected areas and its structural edges. This supports the sensitivity of the network to clinically relevant attributes like lesion shape, texture and variations in contrast. The fact that the high SHAP values are always centralized around the areas of abnormal tissue suggests that the model has learned rather relevant discriminative features that correlate with radiological findings. Such interpretability analysis allows to believe in the reliability of the offered ResNetMSDAM structure and strengthen its transparency and clinical applicability to the automated diagnosis of brain tumor. The SHAP values shown in Fig. 14 are computed on the pooled deep feature representation rather than directly on the pixel space. Therefore, the heatmaps represent the relative importance of learned feature channels instead of spatial pixel-level contributions. Spatial localization of discriminative regions is provided separately through Grad-CAM visualizations. Although the Grad-CAM and SHAP visualizations offer qualitative insights into the model’s decision-making process, quantitative assessment of explainability remains challenging in multi-class medical imaging tasks because of the absence of precise pixel-level ground-truth attribution maps. Hence, the present study focuses on qualitative interpretation instead of quantitative explainability metrics such as localization accuracy and explanation faithfulness.

### Performance comparison with existing methods

**Table 5:** Comparison of the proposed MS-DAM–ResNet101 model with representative brain tumor classification approaches reported in the literature.

Study	Dataset	Classes	Method	Accuracy (%)
Sachdeva et al.^41^	Brain MRI dataset	6	PCA-ANN	91.0
Shyamala et al.^42^	CT + MRI dataset	2	Deep feature fusion	97.0
Proposed MS-DAM-ResNet101	Brain MRI dataset	**15**	Deep CNN + MS-DAM	**99.8**

Table 5 compares the proposed MS-DAM-ResNet101 model with representative brain tumor classification approaches reported in the literature. It should be noted that many previous studies address a limited number of tumor classes (typically 2–6), whereas the proposed framework performs a more challenging 15-class brain tumor classification. Despite the increased classification complexity, the proposed model achieves superior performance, demonstrating its robustness and effectiveness for large-scale multi-class tumor diagnosis.

## Conclusions

This study presents a clinically reliable deep learning framework for multi-class brain tumor classification from Magnetic Resonance Imaging (MRI) scans by integrating a ResNet101 backbone with a Multi-Scale Deformable Attention Module (MS-DAM). The proposed architecture effectively addresses heterogeneous tumor morphology and complex inter-class variations through adaptive multi-scale attention and enriched feature fusion. The model achieved strong diagnostic performance, obtaining validation and test accuracies of 96.89% and 99.21%, respectively, outperforming existing approaches across multiple evaluation metrics, including Accuracy, Precision, Recall, F1-score, Dice coefficient, Specificity, FDR, FOR, FNR, and Cohen’s Kappa. Confusion matrix analysis demonstrated high per-class recognition, with minimal and clinically explainable misclassifications. Robustness experiments confirmed stable performance under Gaussian noise perturbations and up to 50% resolution degradation, supporting practical applicability in real-world clinical settings. ROC and Precision–Recall analyses showed near-perfect separability across all 15 tumor classes (AUC ≈ 1.00). Feature-space visualization via t-SNE revealed well-separated clusters, confirming strong inter-class discrimination in the learned latent space. Interpretability assessments using Grad-CAM and SHAP further demonstrated that the model consistently focuses on clinically relevant tumor regions, enhancing transparency and diagnostic trust. Bootstrap-based confidence interval analysis (95% CI: 98.43%–99.8%) indicated low performance variability and strong reliability across data partitions. Overall, the proposed ResNet101 + MS-DAM framework offers a robust, interpretable, and clinically viable solution for automated multi-class brain tumor classification.

Although the suggested framework has demonstrated great performance, there are a several directions that can be taken up in future research. First, the patient-wise cross-validation experiments can be performed in order to make the reported results even more statistically reliable. Second, assessing the proposed model on multi-institutional or external brain MRI data would be useful in determining its generalization ability across different imaging protocols and clinical settings. Third, the quantitative explainability evaluation metrics which include localization accuracy and explanation faithfulness may be used in future work to complement the current Grad-CAM and SHAP visual interpretations. Finally, integration of the proposed framework into clinical decision-support pipelines and real-time diagnostic systems could further validate its practical applicability in hospital settings.

## Data Availability

The implementation code, training pipeline, and evaluation scripts used in this study are publicly available to ensure transparency and reproducibility of the reported results. The complete source code can be accessed through the following GitHub repository: https://github.com/bathinisiddareddy-arch/siddareddy.

## References

[1] McFaline-Figueroa, J. R. & Lee, E. Q. Brain tumors. *Am. J. Med.* **131**(8), 874–882. 10.1016/j.amjmed.2017.12.039 (2018). 29371158 10.1016/j.amjmed.2017.12.039

[2] DeAngelis, L. M. Brain tumors. *N. Engl. J. Med.* **344** (2), 114–123. 10.1056/NEJM200101113440207 (2001). 11150363 10.1056/NEJM200101113440207

[3] Tandel, G. S. et al. A review on a deep learning perspective in brain cancer classification. *Cancers* **11** (1), 111. 10.3390/cancers11010111 (2019). 30669406 10.3390/cancers11010111 PMC6356431

[4] Zarenia, E., Far, A. A. & Rezaee, K. Automated multi-class MRI brain tumor classification and segmentation using deformable attention and saliency mapping. *Sci. Rep.* **15**, 8114. 10.1038/s41598-025-92776-1 (2025). 40057634 10.1038/s41598-025-92776-1 PMC11890586

[5] Chinga, A., Bendezu, W. & Angulo, A. Comparative study of CNN architectures for brain tumor classification using MRI: Exploring GradCAM for visualizing CNN focus. *Eng. Proc.* **83**(1), 22. 10.3390/engproc2025083022 (2025).

[6] Khawaldeh, S., Pervaiz, U., Rafiq, A. & Alkhawaldeh, R. S. Noninvasive grading of glioma tumor using magnetic resonance imaging with convolutional neural networks. *Appl. Sci. (Basel)* **8**(1), 27. 10.3390/app8010027 (2018).

[7] Sultan, H. H., Salem, N. M. & Al-Atabany, W. Multi-classification of brain tumor images using deep neural network. *IEEE Access* **7**, 69215–69225. 10.1109/ACCESS.2019.2919122 (2019).

[8] Ayadi, A., Elhamzi, W., Charfi, I. & Atri, M. Deep CNN for brain tumor classification. *Neural Process. Lett.* **53**, 671–700. 10.1007/s11063-020-10398-2 (2021).

[9] Deepak, S. & Ameer, P. M. Brain tumor classification using deep CNN features via transfer learning. *Comput. Biol. Med.* **111**, 103345. 10.1016/j.compbiomed.2019.103345 (2019). 31279167 10.1016/j.compbiomed.2019.103345

[10] Çinar, A. & Yildirim, M. Detection of tumors on brain MRI images using the hybrid convolutional neural network architecture. *Med. Hypotheses* **139**, 109684. 10.1016/j.mehy.2020.109684 (2020). 32240877 10.1016/j.mehy.2020.109684

[11] Nayak, D. R., Padhy, N., Mallick, P. K., Zymbler, M. & Kumar, S. Brain tumor classification using Dense Efficient-Net. *Axioms* **11**(1), 34. 10.3390/axioms11010034 (2022).

[12] Srinivas, C. et al. Deep transfer learning approaches in performance analysis of brain tumor classification using MRI images. *J. Healthc. Eng.* **2022**, 3264367. 10.1155/2022/3264367 (2022). 35299683 10.1155/2022/3264367 PMC8923754

[13] Polat, Ö., Dokur, Z. & Ölmez, T. Brain tumor classification by using a novel convolutional neural network structure. *Int. J. Imaging Syst. Technol.* **32** (5), 1646–1660. 10.1002/ima.22763 (2022).

[14] Huang, Z. et al. Convolutional neural network based on complex networks for brain tumor image classification with a modified activation function. *IEEE Access* **8**, 89281–89290. 10.1109/ACCESS.2020.2993618 (2020).

[15] Mondal, A. & Shrivastava, V. K. A novel parametric Flatten-p Mish activation function based deep CNN model for brain tumor classification. *Comput. Biol. Med.* **150**, 106183. 10.1016/j.compbiomed.2022.106183 (2022). 37859281 10.1016/j.compbiomed.2022.106183

[16] Khan, M. A. & Auvee, R. B. Z. Comparative analysis of resource-efficient CNN architectures for brain tumor classification. *In Proceedings of the 2024 27th International Conference on Computer and Information Technology (ICCIT)* (pp. 639–644). IEEE. (2024). 10.1109/ICCIT64611.2024.11021970

[17] Khan, M. A. & Park, H. A convolutional block base architecture for multiclass brain tumor detection using magnetic resonance imaging. *Electronics* **13**(2), 364. 10.3390/electronics13020364 (2024).

[18] Chatterjee, S., Nizamani, F. A., Nürnberger, A. & Speck, O. Classification of brain tumours in MR images using deep spatiospatial models. *Sci. Rep.* **12**, 1505. 10.1038/s41598-022-05572-6 (2022). 35087174 10.1038/s41598-022-05572-6 PMC8795458

[19] Verma, A. & Yadav, A. K. Improved multi-class brain tumor MRI classification with DS-Net: A patch-based deep supervision approach. *Multimed. Tools Appl.* **84**, 36837–36870. 10.1007/s11042-025-20668-7 (2025).

[20] Jia, Q. & Shu, H. BiTr-Unet: A CNN-transformer combined network for MRI brain tumor segmentation. In A. Crimi & S. Bakas (Eds.), *Brainlesion: Glioma, multiple sclerosis, stroke and traumatic brain injuries* (Lecture Notes in Computer Science, Vol. 12963, pp. 3–14). Springer. (2022). 10.1007/978-3-031-09002-8_1 10.1007/978-3-031-09002-8_1 PMC9396958 36005929

[21] Karthik, A. et al. Unified approach for accurate brain tumor multi-classification and segmentation through fusion of advanced methodologies. *Biomed. Signal Process. Control.* **100**(Part A), 106872. 10.1016/j.bspc.2024.106872 (2025).

[22] Rastogi, D., Johri, P., Tiwari, V. & Elngar, A. A. Multi-class classification of brain tumour magnetic resonance images using multi-branch network with inception block and five-fold cross validation deep learning framework. *Biomed. Signal Process. Control.* **88**(Part A), 105602. 10.1016/j.bspc.2023.105602 (2024).

[23] Tabatabaei, S., Rezaee, K. & Zhu, M. Attention transformer mechanism and fusion-based deep learning architecture for MRI brain tumor classification system. *Biomed. Signal Process. Control.* **86**(Part A), 105119. 10.1016/j.bspc.2023.105119 (2023).

[24] Tummala, S., Kadry, S., Bukhari, S. A. C. & Rauf, H. T. Classification of brain tumor from magnetic resonance imaging using vision transformers ensembling. *Curr. Oncol.* **29**(10), 7498–7511. 10.3390/curroncol29100590 (2022). 36290867 10.3390/curroncol29100590 PMC9600395

[25] Mao, Y., Kim, J., Podina, L. & Kohandel, M. Dilated SE-DenseNet for brain tumor MRI classification. *Sci. Rep.* **15**, 3596. 10.1038/s41598-025-86752-y (2025). 39875423 10.1038/s41598-025-86752-y PMC11775108

[26] Akinyelu, A. A., Zaccagna, F., Grist, J. T., Castelli, M. & Rundo, L. Brain tumor diagnosis using machine learning, convolutional neural networks, capsule neural networks and vision transformers, applied to MRI: A survey. *J. Imaging.* **8**(8), 205. 10.3390/jimaging8080205 (2022). 35893083 10.3390/jimaging8080205 PMC9331677

[27] Aamir, M. et al. A deep learning approach for brain tumor classification using MRI images. *Comput. Electr. Eng.* **101**, 108105. 10.1016/j.compeleceng.2022.108105 (2022).

[28] Ghassemi, N., Shoeibi, A. & Rouhani, M. Deep neural network with generative adversarial networks pre-training for brain tumor classification based on MR images. *Biomed. Signal Process. Control.* **57**, 101678. 10.1016/j.bspc.2019.101678 (2020).

[29] Raza, A. et al. A hybrid deep learning-based approach for brain tumor classification. *Electronics* **11**(7), 1146. 10.3390/electronics11071146 (2022).

[30] Asiri, A. A. et al. Optimized brain tumor detection: A dual-module approach for MRI image enhancement and tumor classification. *IEEE Access* **12**, 42868–42887. 10.1109/ACCESS.2024.3379136 (2024).

[31] Anaraki, A. K., Ayati, M. & Kazemi, F. Magnetic resonance imaging-based brain tumor grades classification and grading via convolutional neural networks and genetic algorithms. *Biocybern. Biomed. Eng.* **39**(1), 63–74. 10.1016/j.bbe.2018.10.004 (2019).

[32] Srinivasa Reddy, A. Effective CNN-MSO method for brain tumor detection and segmentation. *Mater. Today Proc.* **57**(Part 5), 1969–1974. 10.1016/j.matpr.2021.10.145 (2022).

[33] Özyurt, F., Sert, E., Avci, E. & Dogantekin, E. Brain tumor detection based on convolutional neural network with neutrosophic expert maximum fuzzy sure entropy. *Measurement* **147**, 106830. 10.1016/j.measurement.2019.07.058 (2019).

[34] Sajjad, M. et al. Multi-grade brain tumor classification using deep CNN with extensive data augmentation. *J. Comput. Sci.* **30**, 174–182. 10.1016/j.jocs.2018.12.003 (2019).

[35] Kaplan, K., Kaya, Y., Kuncan, M. & Ertunç, H. M. Brain tumor classification using modified local binary patterns (LBP) feature extraction methods. *Med. Hypotheses.* **139**, 109696. 10.1016/j.mehy.2020.109696 (2020). 32234609 10.1016/j.mehy.2020.109696

[36] Khalil, H. A., Darwish, S., Ibrahim, Y. M. & Hassan, O. F. 3D-MRI brain tumor detection model using modified version of level set segmentation based on dragonfly algorithm. *Symmetry* **12**(8), 1256. 10.3390/sym12081256 (2020).

[37] Alam, M. S. et al. Automatic human brain tumor detection in MRI image using template-based K means and improved fuzzy C means clustering algorithm. *Big Data Cogn. Comput.* **3**(2), 27. 10.3390/bdcc3020027 (2019).

[38] Rajesh Babu, K., Nagajaneyulu, P. V. & Satya Prasad, K. Brain tumor segmentation of T1w MRI images based on clustering using dimensionality reduction random projection technique. *Current Medical Imaging Formerly Current Medical Imaging Reviews* **17**(3), 331–341. 10.2174/1573405616666200712180521 (2021). 10.2174/1573405616666200712180521 32652918

[39] Huang, H., Meng, F., Zhou, S., Jiang, F. & Manogaran, G. Brain image segmentation based on FCM clustering algorithm and rough set. *IEEE Access* **7**, 12386–12396. 10.1109/ACCESS.2019.2893063 (2019).

[40] Nagah Hennes, W. Brain tumor for 14 classes [Data set]. Kaggle. (2023). https://www.kaggle.com/datasets/waseemnagahhenes/brain-tumor-for-14-classes

[41] Sachdeva, J., Kumar, V., Gupta, I., Khandelwal, N. & Ahuja, C. K. Segmentation, feature extraction, and multiclass brain tumor classification. *J. Digit. Imaging.* **26**(6), 1141–1150. 10.1007/s10278-013-9600-0 (2013). 23645344 10.1007/s10278-013-9600-0 PMC3824920

[42] Shyamala, N. & Basha, S. M. Multi-modal deep feature extraction and classifier-level integration for brain tumour classification using CT and MRI image. *Artif. Intell. Eng.*10.1049/aie2.70009 (2026).

